# Initiation of breastfeeding within one hour of birth and its determinants among normal vaginal deliveries at primary and secondary health facilities in Bangladesh: A case-observation study

**DOI:** 10.1371/journal.pone.0202508

**Published:** 2018-08-16

**Authors:** Farhana Karim, Sk. Masum Billah, Mohiuddin Ahsanul Kabir Chowdhury, Nabila Zaka, Alexander Manu, Shams El Arifeen, Abdullah Nurus Salam Khan

**Affiliations:** 1 Maternal and child health division, International Centre for Diarrhoeal Disease Research, Bangladesh (icddr,b), Dhaka, Bangladesh; 2 Health Section, Maternal and Newborn Health, UNICEF, New York, New York, United States of America; 3 Centre for Maternal and Newborn Health, Liverpool School of Tropical Medicine, Liverpool, United Kingdom; Indiana University School of Medicine, UNITED STATES

## Abstract

**Background:**

Initiation of breastfeeding within one hour of birth can avert 22% of newborn mortality. Several factors influence breastfeeding practice including mothers’ socio-demographic and obstetric characteristics, and factors related to time around child birth. This study explores breastfeeding initiation practices and associated influencing factors for initiating breastfeeding within one hour of birth in public health facilities of Bangladesh.

**Methods:**

In this study, normal deliveries were observed in 15 public health facilities from 3 districts in Bangladesh. Study participants were selected by convenient sampling i.e. delivery cases attending health facilities during the study period were selected excluding caesarean section deliveries. Among 249 mothers, time of initiation of breastfeeding was observed and its association was measured with type of health facility, privacy in delivery room, presence of separate staff for newborn, spontaneous breathing, skin-to-skin contact and postnatal contact of mother or newborn with health care providers within one hour after delivery. Data was collected during August-September, 2016. Kruskal-Wallis test was used to measure equality of median duration of breastfeeding initiation time among two or more categories of independent variables. Series of simple logistic regressions were conducted followed by multiple logistic regression to identify the determinants for breastfeeding initiation within one hour.

**Results:**

Among 249 mothers observed, 67% initiated breastfeeding within one hour of birth at health facilities and median time to initiate breastfeeding was 38 minutes (Inter-quartile range: 20–56 minutes). After controlling for maternal age as potential confounder, the odds of initiating breastfeeding within one hour of birth was significantly higher if mothers gave birth in district hospitals (AOR 3.5: 95% CI 1.5, 6.4), visual privacy was well-maintained in delivery room (AOR 2.6: 95% CI 1.2, 4.8), newborns cried spontaneously (AOR 4.9: 95% CI 3.4, 17.2), were put to skin-to-skin contact with mothers (AOR 3.4: 95% CI 1.9, 10.4) or were examined by health care providers in the facilities (AOR 2.4: 95% CI 1.3, 12.9).

**Conclusions:**

In health facilities, initiation of breastfeeding within one hour is associated with some critical practices and events around the time of birth. With the global push toward facility-based deliveries, it is very important to identify those key factors, within the landscape of maternal and newborn care, which significantly enable health care providers and parents to engage in the evidence-based newborn care activities including early initiation of breastfeeding that will, in turn, reduce global rates of newborn mortality.

## Background

Initiation of breastfeeding after birth is an integral part of the safe delivery procedure [1]) and is widely acknowledged as a beneficial practice. Lancet neonatal survival series identifies breastfeeding as one effective intervention that can reduce 55–87% of all-cause neonatal mortality and morbidity [2]. Several studies find that breastfeeding reduces the risk of neonatal deaths particularly due to infections [3, 4] like diarrhoea [5, 6], neonatal sepsis [7, 8], pneumonia and meningitis [9]. When further explored, delayed initiation of breastfeeding was found increasing the mortality risks among newborns [10]. Recent evidence shows, newborns who were put to breast within one hour of birth had 29% less chance of dying within the first 28 days of their lives than those who were breastfed 2–23 hours of birth [11]. Initiation of breastfeeding within one hour of birth can also avert up to 22% of all newborn deaths [12, 13] and the recent Lancet Every Newborn series mentions that the mortality reduction can reach up to 44% [12–14].

The World Health Organization (WHO), therefore, recommends that breastfeeding should be initiated early and preferably within one hour of birth [15]. In recent years much efforts are being made by national and international stakeholders to encourage mothers to initiate breastfeeding early [16]. Yet, only 45% of world’s newborn and 42% of newborns in South-Asia are put to breast within one hour of birth [17]. The most recent demographic health surveillance report in Bangladesh shows that only a half of the mothers start breastfeeding within one hour of birth [18]. Interestingly, the trend of early breastfeeding initiation, identified within the last decade from similar surveys, shows that mothers are less likely to practice so if they give birth in health facility than at home [18–20]. This persistent lower coverage of early breastfeeding initiation within health facilities is of concern since promoting institutional delivery (a proxy for skilled attendance) is a priority intervention to reach the targets of Sustainable Development Goals (SDG) of reducing maternal and child mortality by 2030 [21]. In line with the increasing institutional delivery rates in the country [18] determining the factors that influence early initiation of breastfeeding in health facility is a public health imperative to improve this life saving practice.

Several factors influence breastfeeding practice and its impact varies across different regions and sub-populations of the world. Mothers’ socio-demographic and obstetric characteristics, exposure to health care support services and also the existing community beliefs principally influence the timing of breastfeeding initiation especially in the South Asian countries [22–25]. While these characteristics are well explored in several studies to find their association with early initiation of breastfeeding, evidences remain limited for characteristics that are related to time around child birth. Such characteristics including place of delivery and its breastfeeding friendly environment, mode of delivery, post-partum health condition of mother and newborn, support and guidance provided by the birth attendants and family members are found to influence the timing of breastfeeding initiation after delivery [26–29]. In most cases, these factors were explored either from hospital records or recall based surveys of mothers after delivery. In countries with poor record keeping system, it is difficult to determine facility based practices conducted around the time of birth from hospital records [30] and recall based surveys limit the data validity due to poor recollection of birth events and breastfeeding experiences by the mothers [12, 31]. This study, based on direct observation of activities around the time of birth, aims to generate accurate and valid evidences for identifying associated factors influencing initiation of breastfeeding within one hour of delivery in health facilities. The study also generates actionable-evidences to direct policy makers to identify and prioritize scopes for creating and supporting breastfeeding friendly environment for mothers delivering in health facilities.

## Methodology

### Study design

Government of Bangladesh, with support from UNICEF headquarter and UNICEF Bangladesh, is implementing the “Every Mother Every Newborn Quality Improvement (EMEN QI)” initiatives in selected government health facilities to improve care around the time of childbirth. A baseline evaluation was conducted prior to implementing EMEN QI standards in twelve sub-district hospitals and three district hospitals of the three neighbouring districts–Kurigram, Lalmonirhat and Gaibandha, situated in northern part of Bangladesh during August-September, 2016. This paper draws the findings from the baseline evaluation that majorly employed hospital based direct observation to identify quality of intrapartum and immediate postpartum care. Sample size was originally calculated based on the aforementioned objectives of the baseline study and the target was to observe 500 delivery cases assuming 50% prevalence of the quality of care indicators and design effect of 1.5. Convenient or non-probability sampling method was applied i.e. delivery cases attending the selected facilities during the study period were selected as potential study participants. In selection of participants, deliveries conducted by caesarean section were excluded as this is a known barrier to early initiation of breastfeeding [28, 32, 33].

### Definitions and ascertainment of outcome and explanatory variables

#### Outcome variable

Initiation of breastfeeding within one hour was considered as the outcome variable in this study.

#### Explanatory variables

Based on the literature review, we considered a number of factors as the explanatory variables which could potentially influence the time to initiate breastfeeding after birth in health facility [28, 34, 35]. These included type of health facility, privacy in delivery room, presence of separate staff for newborn care, spontaneous breathing or crying of newborn, skin-to-skin contact with mother, and newborn receiving a postnatal examination within one hour of delivery. Mother’s socio-demographic characteristics such as age, education, occupation, religion and obstetric characteristics such as parity and gestational age at the time of delivery were also considered as the individual level explanatory variables. Operational definitions for the explanatory variables under consideration are summarized in Table 1.

**Table 1:** The explanatory variables and their operational definitions.

Variables	Operational definitions
**Maternal characteristics**	
Maternal age	Mother’s age at time of delivery
Maternal education	Mother’s highest level of education
Maternal occupation	Mother’s occupation status at time of delivery
Maternal religion	Muslim Non- Muslim
Parity	Primiparous (first time having child birth); Multiparous (having childbirth more than once)
Gestational age	Preterm pregnancy (birth before 37 completed weeks); Term pregnancy (birth at or after 37 completed weeks)
**Characteristics related to birth events or time around birth**	
Type of health facility	District hospital (secondary level health care facility); Upazila health complex (primary level health care facility)
Privacy in delivery room	The visual privacy is maintained for individuals either through partition or curtain between beds
Separate staff for newborn	Presence of separate staff along with the birth attendant for taking care of the newborn
Spontaneous breathing or crying of newborn	The newborn spontaneously breathes or cries after birth without any basic or advanced resuscitation support
Skin to skin contact	The baby is put skin-to-skin on mother’s bare abdomen or chest
Postnatal consultation or contact with mother and newborn within one hour of delivery	Physical examination of the newborn or mother or any kind of post-natal contact within one hour of delivery

#### Data collection method for ascertainment of variables

The study employed direct observation of labour and immediate newborn care practices using a validated observation tool developed by Maternal and Child Health Integrated Program (MCHIP) [36] and used in similar observation based study [37]. National standard operating procedure (SOP) for maternal care [38] was consulted for further contextualization of the observation tool (S1 Appendix). Breastfeeding initiation time was determined from the interval between two key events–giving live birth and initiation of breastfeeding. Time of live birth was recorded as soon as the baby was completely delivered and time of initiation of breastfeeding was recorded when the baby was first put to mother’s breast for feeding. In cases where one obstetric case was observed by more than one observer, the same watch was used throughout the observation period for keeping track of the key events to avoid inter-observer variation in recording time. The times were recorded in completed hours and minutes. A maximum observation time was limited to 24 hours since the time of birth because within this time period most of the mothers (about 90%) were likely to initiate breastfeeding according to the recent demographic survey [18]. However, the observation ended early when a mother was discharged from the facility before 24 hours of delivery.

The observation also explored the environment of labour rooms including maintenance of the privacy and presence of separate staff for newborn care at time of delivery. Information on recently delivered mothers’ age, education, occupation, religion and obstetric characteristics such as parity and gestational age at the time of delivery were obtained by interviewing the women after delivery using a structured interview questionnaire (S2 Appendix). Informed written consent was sought from every woman before interview. For delivery observation informed written consent was sought from the birth attendants and verbal consent was taken from each participant. In case of participants under age 18, written consent was taken from legal guardians.

### Data collection

**Fig 1:**
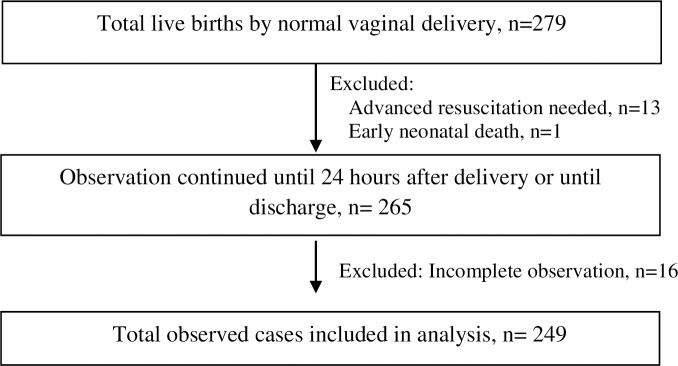
Selection of study participants for data collection and analysis.

A team of twenty (20) trained research physicians initiated the structured observation of intrapartum and postpartum activities when the recently delivered mothers attended the selected health facilities for normal vaginal delivery (NVD) during August-September, 2016. Preference was given to females in selecting research physicians since it was culturally more acceptable for them to directly observe deliveries in Bangladesh context. The research physicians maintained a round the clock roster during the data collection period to continue observation without interruptions. To minimize inter-observer bias, all twenty research physicians were trained by expert obstetricians on the structured observation checklist followed by two days of facility based practice in pairs. This ensured a standardized ascertainment of both outcome and explanatory variables. A total of 279 NVD cases were observed, however, observation did not continue for one early newborn death and 13 newborns needing advanced resuscitation support (Fig 1). A total of 16 cases (6%) had incomplete information on observation thus leaving 249 cases to be included in the final analysis (Fig 1).

### Statistical analysis

The simple descriptive statistics such as percentage for categorical variables were employed to see the distribution of samples according to socio-demographic characteristics. Distributions of the time intervals between delivery and breastfeeding initiation were checked for normality. As the time intervals were not normally distributed, median time to initiate breastfeeding stratified by possible covariates was estimated along with interquartile range (IQR). Kruskal-Wallis test was carried out for testing the equality of medians among the categories of the explanatory variables. A series of simple logistic regressions was conducted with all the potential covariates to identify the determining factors for initiation of breastfeeding within one hour in a health facility. Finally, multiple logistic regression was conducted including the variables found significant at simple logistic regressions. Maternal age was considered as potential confounder for adjusting in the regression model. Individual covariate effects on the outcome variable were indicated by the adjusted odds ratio (AOR). P-value less than 0.05 is considered as statistically significant for all the tests conducted. The analyses are performed using Stata 13.0.

## Results

### Socio-demographic and obstetric characteristics of mothers

A total of 249 live births by NVD were observed. Mean (±SD) age of the mothers was 23.5 (±0.3) years and majority of the mothers aged between 15 to 24 years (60%) (Table 2). Only 30% of the mothers completed the secondary level of education. Majority were Muslim (90%) and were housewives (92%). About three-fourth of the mothers completed 37 weeks of pregnancy at the time of their delivery and half of them was having childbirth for the first time (primiparous).

**Table 2:** Socio-demographic and obstetric characteristics of the recently delivered mothers, n = 249.

Characteristic of recently delivered mothers, n = 249	Frequency	Percentage
Age (in years)		
15–24	149	59.8
25–34	87	34.9
35 and above	13	5.2
Highest level of education		
No formal education or primary incomplete	71	28.5
Primary complete	105	42.2
Secondary complete or higher	73	29.3
Occupation		
Unemployed/ Housewife	229	92.0
Employed	20	8.0
Religion		
Muslim	225	90.4
Non-Muslim	24	9.6
Gestational age at time of delivery		
Pre-term pregnancy (< 37 weeks)	23	9.2
Term pregnancy (≥ 37 weeks)	188	75.5
Missing information	38	15.3
Parity		
Primiparous	126	50.6
Multiparous	120	48.2
Missing information	3	0.2

### Time of initiation of breastfeeding

Table 3 summarizes the median time to initiate breastfeeding across different maternal and facility level characteristics. Overall, median time to initiate breastfeeding was 38 minutes. There were no significant differences in the median time to initiate breastfeeding among different categories of mother’s age, education, occupational status, and religion. Similarly the initiation time did not differ between the mothers who delivered at full term pregnancy and those who had a pre-term delivery and also between the mothers who were first time pregnant i.e. primiparous and those who were multiparous. However, delivering in a sub-district hospital significantly delayed the breastfeeding initiation in comparison to delivering in a district hospital (43 vs. 30 minutes). Maintenance of adequate privacy in the delivery room and presence of separate staff for taking care of newborn significantly reduced the time in early breastfeeding initiation. Initiation time was significantly longer if the newborns needed resuscitation support for delayed breathing (50.5 vs. 37 minutes, p = 0.01). However, initiation time was significantly early if babies were put skin-to-skin on their mother’s abdomen (32 vs. 40 minutes, p = 0.001) and had postnatal contact with health care provider either during postnatal examinations of newborn or consultation with mothers within one hour of birth (21.5 vs. 40 minutes, p = 0.001).

**Table 3:** Distribution of median time to initiate breastfeeding by different maternal and facility level characteristics, n = 249.

Characteristics of recently delivered mothers	Median time to initiate breastfeeding (minutes)	Inter-quartile range (minutes)	P value ^a^
**Total**	**38**	**20–56**	**-**
Age (in years)			
15–24	38	20–57	0.885
25–34	38	22–55	
35 and above	38	35–52	
Highest level of education			
No formal education or primary incomplete	35	20–50	0.439
Primary complete	40	20–61	
Secondary complete or higher	39.5	26–57	
Occupation			
Unemployed / housewife	38	22–55	0.787
Employed	37.5	20–56	
Religion			
Muslim	39.5	23–57	0.128
Non-Muslim	30	20–40	
Gestational age at time of delivery			
Pre-term pregnancy (< 37 weeks)	40	25–55	0.587
Term pregnancy (≥ 37 weeks)	38	24–60	
Parity			
Primiparous	37	20–57	0.537
Multiparous	39	24–55	
Type of facility			
District hospitals	30	16.5–40	0.001
Sub-district hospitals	43	30–70	
Privacy in delivery room			
Maintained	30	19–45	0.001
No privacy	44	34–71.5	
Separate staff for newborn care			
At least one staff present	40	30–60	0.009
None	35	19–53	
Newborn breathing or crying			
Spontaneous	37	20–51	0.01
Delayed/after resuscitation	50.5	27.5–78	
Skin to skin contact			
Yes	32	15–40	0.001
No	40	24–68	
Postnatal contact with newborn or mother within one hour of delivery			
Yes	21.5	15–35	0.001
No	40	27–60	

^a^ Kruskal-Wallis test.

### Determinants of initiating breastfeeding within one hour of birth

During the total observation period, 171 women of the total 249 observed cases (68.7%) initiated breastfeeding within one hour of birth (data not shown in table). Table 4 summarizes the unadjusted and adjusted odds ratios from the simple and multiple logistic regression model, respectively, for the study outcome i.e. initiation of breastfeeding within one hour of birth. After controlling for maternal age as a potential confounder, the odds of initiating breastfeeding within one hour of birth was found higher if mother gave birth in district hospital than in sub-district hospital (AOR 3.5: 95% CI 1.5, 6.4), and in delivery room where visual privacy was well-maintained than no privacy (AOR 2.6: 95% CI 1.2, 4.8). The babies who breathed or cried spontaneously after birth had nearly 5 times odds of being breastfed within one hour to that among those who needed resuscitation (AOR 4.9: 95% CI 3.4, 17.2). The practice of putting the baby skin-to-skin on mother’s abdomen (AOR 3.4: 95% CI 1.9, 10.4) and postnatal contact with any health care provider within one hour of birth in the facility (AOR 2.4: 95% CI 1.3, 12.9) were both found to be strong predictors for early initiation of breastfeeding.

**Table 4:** Factors associated with breastfeeding initiation within one hour of birth, from logistic regression of 249 observed cases.

Variable	Total	% of mothers initiating breastfeeding within 1 hour	UOR	95%CI	P value	AOR	95% CI	P value
Age (in years)								
15–24	149	66.4	1.0	**-**	**-**	1.0	**-**	**-**
25–34	87	71.3	1.3	0.7–2.2	0.443	0.9	0.6–2.8	0.359
35 and above	13	76.9	1.7	0.4–6.4	0.444	1.4	0.6–18.2	0.151
Highest level of education								
No formal education or primary incomplete	71	69.0	1.0	**-**	**-**			
Primary complete	105	68.6	0.98	0.5–1.9	0.950			
Secondary complete or higher	73	68.5	0.98	0.5–2.0	0.946			
Occupation								
Unemployed / housewife	229	68.6	1.0	**-**	**-**			
Employed	20	70.0	1.1	0.4–2.9	0.894			
Religion								
Muslim	24	83.3	1.0	-	**-**			
Non-Muslim	225	67.1	0.4	0.1–1.2	0.113			
Gestational age at time of delivery								
Pre-term pregnancy (< 37 weeks)	126	65.1	1.0	**-**	**-**			
Term pregnancy (≥ 37 weeks)	120	71.7	1.4	0.8–2.3	0.267			
Type of facility								
District hospitals	114	82.5	3.5	2.0–6.4	0.0001	3.2	1.5–6.4	0.002
Sub-district hospitals	135	57.0	1.0	**-**	**-**	1.0	**-**	**-**
Privacy in delivery room								
Maintained	161	77.0	2.9	1.7–5.1	0.0001	2.6	1.2–4.8	0.010
No privacy	88	53.4	1.0	**-**	**-**	1.0	**-**	-
Separate staff for newborn								
At least one staff present	90	65.6	0.80	0.5–1.4	0.460			
None	159	70.4	1.0	-				
Newborn breathing or crying								
Spontaneous	198	77.8	7.0	3.6–13.7	0.0001	4.9	3.4–17.2	0.000
Delayed/ after resuscitation	51	33.3	1.0	**-**	**-**	1.0	**-**	-
Skin to skin contact								
Yes	76	88.2	4.9	2.3–10.6	0.0001	3.4	1.9–10.4	0.001
No	173	60.1	1.0	**-**	**-**	1.0	**-**	-
Postnatal contact with newborn or mother within one hour of delivery								
Yes	47	87.2	3.8	1.5–9.3	0.0012	2.4	1.3–12.9	0.015
No	202	64.4	1.0	**-**	**-**	1.0	**-**	-

UOR: Unadjusted Odds Ratio; AOR: Adjusted Odds Ratio, adjusted for maternal age only; CI: Confidence Interval.

## Discussion

Despite the universal recommendation of early initiation of breastfeeding, our study finds only seven out of every ten newborns are breastfed within one hour of birth in health facilities. The observed proportion of this practice among the normal vaginal deliveries is much higher than the reported average among all facility deliveries in Bangladesh (38%) and also of some other south-east Asian countries—Pakistan (16%), India (34%) and Nepal (56%) as stated in their most recent demographic health surveillance (DHS) reports [18, 39–41]. A possible reason for this marked difference between observed and reported proportions is that the DHS report accounts for the facility deliveries by caesarean sections, which is a known barrier to early initiation of breastfeeding [33, 42, 43]. The multiple regression model of the study finds some critical practices taking place at time around the birth facilitate the introduction of early breastfeeding. These practices include putting a newborn to mother’s abdomen immediately after birth for skin to skin contact, maintenance of privacy in delivery room, spontaneous breathing or crying and receiving postnatal examination of the baby within one hour in the facility. The findings emphasize the need for promoting these practices for an optimal breastfeeding friendly environment in the facility since the inception of mother in labour till her discharge from the facility.

Keeping the mother and newborn together is a prerequisite to initiate the breastfeeding early as their separation, even for a very brief period, results in delayed breastfeeding initiation [44]. Our study observed that the practice of putting the baby skin-to-skin on mother’s abdomen significantly reduced the breastfeeding initiation time, which is consistent with findings from several other studies [34, 45, 46]. Skin-to-skin contact with mother facilitates early initiation of breastfeeding through release of ‘prolactin’ hormone that promotes lactation and ‘oxytocin’ hormone that promotes attachment to the mother [47]. Therefore the practice is widely promoted by the WHO and UNICEF as part of the immediate newborn care package as it creates an optimal environment for breastfeeding the newborn [48]. In postpartum period, a skilled birth attendant can play the first role to establish the mother-newborn connection through skin-to-skin contact and therefore facilitate the mothers to breastfeed early [49]. Several other factors like lack of privacy and inadequate rooming-in decreases the effectiveness of skin-to-skin practice [50] and therefore can delay the breastfeeding initiation time. This study also finds that maintenance of privacy in the delivery area was one influential factor for early initiation of breastfeeding. Further analysis showed that privacy was significantly better maintained in district hospital (p = 0.001; data not presented in table) where the breastfeeding initiation was also earlier than the sub-district hospital. Therefore, overcoming the circumstantial challenges by provision of optimal environment for mother with motivation from the health care providers should be the first priority to promote skin-to-skin practice and thereby facilitate early breastfeeding in a health facility [51].

Our study found, babies who cried or breathed spontaneously had significantly shorter breastfeeding initiation time than the babies who did not. In the absence of very close monitoring by skilled providers, it is often not advisable to recommend early breastfeeding, until infant's breathing has been stabilized [52, 53]. However, that resuscitation should be judicious and each step should be appropriately timed and ordered to avoid further delay in initiating breastfeeding [35]. Ensuring the timeliness and quality of care during immediate postpartum period need filling up of the resource gap with adequate numbers of trained and skilled personnel especially for complicated newborn management [54]. An integrated package of training like ‘Helping Babies Breathe (HBB)’ by American Academy of Paediatrics can provide a timed step-wise algorithm for neonatal resuscitation that also showed evidence of improved immediate newborn care practices in low-resource settings [55, 56]. In addition, periodic on job training and refresher training of the staff are mandatory for standardized implementation of the evidence based management guidelines.

Postnatal examination of newborn within one hour is another critical window of opportunity to come in contact with a health care provider and was found as a significant predictor for early initiation of breastfeeding in our study. Evidence suggests that a skilled and properly trained health worker can motivate mothers to initiate early breastfeeding and explain its advantages, counsel on dangers of pre-lacteal feeding and its long term risk, and the benefits of exclusive breastfeeding for first six months [57, 58]. Beyond the opportunity to receive counselling on early breastfeeding, the postnatal contact with the health care provider offers a chance of seeking advice regarding any lactational or psychological difficulties experienced by the mothers [59]. One study showed that before giving birth and afterwards, health care providers influenced mother’s feeding decisions at the key moments [60]. In another study, successful breastfeeding was found significantly associated with the satisfaction of mothers regarding the medical care received in health facilities [61]. Health system strengthening thus seeks more attention to ensure routine postnatal care immediately after birth by staff trained on lactation and related complications management.

## Strengths and limitations

Our study assessed the time to initiate breastfeeding after birth in health facilities and associated factors based on actual observations, which is considered as gold standard method to evaluate any health activity [62]. Recording precise time for each key event of the study also provided accurate measurement of time intervals for breastfeeding initiation. However, the findings are not generalizable to all deliveries taking place in health facilities as we only observed normal vaginal deliveries and deliberately excluded the labour cases that needed caesarean section or advanced neonatal care support. As both these conditions are known factors for delayed initiation of breastfeeding, therefore, for preterm and low-birth weight babies, and for babies born by caesarean section, specific exploration of associated factors for breastfeeding initiation would be required. We observed the normal deliveries in health facilities that were equipped to manage complicated obstetric cases, hence the predictors of early initiation of breastfeeding in lower tiers of health facilities where deliveries are conducted by single paramedic could be different than our study findings. Furthermore, since the study design is primarily based on the objectives of the baseline evaluation of a quality improvement initiative for maternity care in health facilities, we could not explore some key factors such as socio-economic condition of the mothers, family and paternal support, knowledge on breastfeeding, or history of antenatal care seeking as they were not explored during data collection.

## Conclusions

While developing countries are progressing towards increasing institutional births, the demand for optimal environment for breastfeeding in the health facilities is also increasing. This study identifies that success in early breastfeeding initiation in a health facility depends more on some critical post-partum factors rather than mother’s individual characteristics. To address these factors, a global awareness on Baby Friendly Hospital Initiative (BFHI) is launched in 1991 by the WHO and UNICEF to promote breastfeeding in maternity wards of the health facilities [63]. One of its mandates is to implement *Ten Steps to Successful Breastfeeding* which requires a holistic approach of improvement in infrastructural and policy environment of the health facilities. This study made an important contribution to the existing evidence-base, is the finding that, in addition to provision of privacy, having staff dedicated to the care of the baby, within the first hour after delivery, is a significant enabling factor for timely initiation of breastfeeding. Context appropriate and innovative platforms to promote skin-to-skin care, to minimize the mother-newborn separation and to support mothers in overcoming the lactation problems should be the priority research areas to promote early initiation of breastfeeding.

## Supporting information

S1 Appendix Observation checklist for delivery care and essential newborn care. (DOCX)

S2 Appendix Exit interview of women and family members. (DOCX)
