# Supplementary material for: Initiation of breastfeeding within one hour of birth and its determinants among normal vaginal deliveries at primary and secondary health facilities in Bangladesh: A case-observation study
Source: PLoS One. 2018 Aug 16;13(8):e0202508. doi: 10.1371/journal.pone.0202508 (PMC6095597; doi:10.1371/journal.pone.0202508)
Supplement: S1 Appendix — (DOCX) [file pone.0202508.s001.docx]

**S1 Appendix: Observation checklist for delivery care and essential newborn care**

**Objective of the observation:**

To assess the quality of service delivery during management of labour, immediate newborn care, postnatal care within 24 hours (before discharge from the facility) and sick newborn management in intervention and comparison facilities.

**Instruction for the data collectors:**

- The data will be collected principally by observation
- If the data collectors face difficulty in collecting information regarding any specific indicator, they will talk to facility managers and/or review documents or talk to the facility manager or any person nominated by him
- The data collectors should pay specific attention to the **SKIP** questions
- If time of event is unknown, record 99:99.
- If any numerical record is unknown record “99” as appropriate in the box.

| **Information about observer** | | | | | |
| --- | --- | --- | --- | --- | --- |
| Name of the observer: ____________________________________ | | | | | Code: |
| Date of observation: | | | --  D D M M Y Y Y Y | | |
| Time of **starting** observation: | **:**  **h h m m** | Time of **ending** observation: | | **:**  **h h m m** | |

| **Information about health facility:** | | | | |
| --- | --- | --- | --- | --- |
| Name of health facility | _____________________________ | |  | |
| Address of the facility: | | | | |
| District __________________________ | | Upazila _________________________ | | |
| Type of the facility | District hospital/ Medical College and hospital | | | 1 |
|  | Upazila Health Complex | | | 2 |
| Facility Ownership | Government | | | 1 |
|  | Private | | | 2 |
|  | NGO | | | 3 |
|  | Others(specify)  _______________________________________ | | | 7 |
| Facility has special arrangement for disabled at reception/ emergency | Yes | | | 1 |
|  | No | | | 2 |
| Facility has ambulance access to reception/ emergency | Yes | | | 1 |
|  | No | | | 2 |

| **Information about the health worker** | | |
| --- | --- | --- |
| *In case of a new health worker (HW) or respondent: Before observing the consultation, make sure to obtain permission from both the HW. Also make sure that the HW knows that you are not there to evaluate him or her, and that you are not an “expert” to be consulted during the session.* | | |
|  | **Options** | **Code** |
| Name | **__________________________________________** |  |
| Age (in years) |  |  |
| Work experience (in years) |  |  |
| Sex | Male | 1 |
|  | Female | 2 |
| Type of provider | Medical officer | 1 |
|  | Nurse | 2 |
|  | FWV | 3 |
|  | SACMO | 4 |
|  | CHCP | 5 |
|  | HA | 6 |
|  | FWA | 7 |
|  | Others(specify)  ____________________________________________ | 8 |

| **Information about the client or pregnant woman** | | |
| --- | --- | --- |
| *After reading the consent form to the client, if she gives the permission, please start observation.*  *If client is incapacitated, family friend/relative/neighbour accompanying client may give consent. (Consent for client cannot be given by health worker or facility in charge. Consent from client herself or her family friend/relative/neighbour must be obtained prior the observation)* | | |
| 01 | Client name | ------------------------------------------------ |
| 02 | Client registration number |  |
| 03 | Age *(in years)* |  |
| 04 | Gestational age *(in weeks)* |  |
| 05 | LMP | **-****-**  **D D \|M M \| Y Y Y Y** |
| 06 | EDD | **-****-**  **D D \|M M \| Y Y Y Y** |
| 07 | Parity |  |
| 08 | Gravida |  |
| 09 | Who gave consent | Client herself 1 |
|  |  | Relative 2 |
|  |  | Family friend/ neighbour 3 |
|  |  | Other(specify)  _________________________ 7 |
| 10 | Time of arrival of client  *(Record in 24 hours)* | **:**  **h h m m** |
| 11 | Day of the week | Sunday 1 |
|  |  | Monday 2 |
|  |  | Tuesday 3 |
|  |  | Wednesday 4 |
|  |  | Thursday 5 |
|  |  | Friday 6 |
|  |  | Saturday 7 |
| 12 | Client coming from | Home/ Someplace else 1 |
|  |  | Other heath facility 2 |
|  |  | ANC ward at this facility 3 |
| 13 | Time of first professional HW contact  *(Record in 24 hours)* | **:**  **h h m m** |

| 14 | Client is admitted for | Labor | 1 | SKIP TO **SECTION 1** |
| --- | --- | --- | --- | --- |
|  |  | Complication | 2 |  |
| 15 | Type of complication | Post-partum haemorrhage | 1 | SKIP TO **SECTION 8** |
|  |  | Pre eclampsia/ eclampsia | 2 | SKIP TO **SECTION 9** |
|  |  | Both | 3 | Skip to **SECTION 8 & 9** |

**Section 1: Initial Client Assessment**

| **No** | **Questions and filters** | | | | **Options/Code** | | | | | **Skip** |
| --- | --- | --- | --- | --- | --- | --- | --- | --- | --- | --- |
|  |  |  |  |  | **Yes** | | | **No** | |  |
| 100 | Is this section observed? | | | | 1 | | | 2 | | Yes 🡪102 |
| 101 | *If No, write down the reason* |  | | | | | | | | Skip to  **Section 2** |
| *Record whether the provider carried out the following steps and/or examinations:*  *(some of the following steps may be performed simultaneously or by more than one provider)* | | | | | | | | | | |
| **Introduction and History Taking** | | | | | | | | | | |
| **No** | **Questions and filters** | | | | **Options/Codes** | | | | | **Skip** |
|  |  |  |  |  | **Yes** | | **No** | | **DK** |  |
| 102 | Respectfully greets woman | | | | 1 | | 2 | | 8 |  |
| 103 | Encourages the women to have a support person present during labour and birth | | | | 1 | | 2 | | 8 |  |
| 104 | Asks woman(or support person) if she has any question | | | | 1 | | 2 | | 8 |  |
| 105 | Checks woman’s health card/ ANC card | | | | 1 | | 2 | | 8 |  |
| 106 | Asks client | | | |  | |  | |  |  |
|  | 1. Age | | | | 1 | | 2 | | 8 |  |
|  | 1. Length of pregnancy | | | | 1 | | 2 | | 8 |  |
|  | 1. Parity/ Gravida | | | | 1 | | 2 | | 8 |  |
| 108 | Asks if she has any of following symptoms for **CURRENT PREGNANCY** | | | | | | | | |  |
|  | 1. Vaginal bleeding | | | | 1 | | 2 | | 8 |  |
|  | 1. Fever | | | | 1 | | 2 | | 8 |  |
|  | 1. Severe headaches | | | | 1 | | 2 | | 8 |  |
|  | 1. blurred vision | | | | 1 | | 2 | | 8 |  |
|  | 1. Swollen face or hands | | | | 1 | | 2 | | 8 |  |
|  | 1. Convulsions | | | | 1 | | 2 | | 8 |  |
|  | 1. loss of consciousness | | | | 1 | | 2 | | 8 |  |
|  | 1. Severe difficulty breathing | | | | 1 | | 2 | | 8 |  |
|  | 1. Severe abdominal pain | | | | 1 | | 2 | | 8 |  |
|  | 1. Decrease or stop in fetal movement | | | | 1 | | 2 | | 8 |  |
|  | 1. If client is concerned about any other problem | | | | 1 | | 2 | | 8 |  |
| 109 | Client has previous pregnancy? *(Observe, listen, or ask)* | | | | 1 | | 2 | |  | No🡪111 |
| 110 | Asks if she had any of following complication during **PREVIOUS PREGNANCIES** | | | | | | | | |  |
|  | 1. Heavy bleeding during or after delivery | | | | 1 | | 2 | | 8 |  |
|  | 1. Anemia | | | | 1 | | 2 | | 8 |  |
|  | 1. High blood pressure | | | | 1 | | 2 | | 8 |  |
|  | 1. Convulsions | | | | 1 | | 2 | | 8 |  |
|  | 1. Multiple pregnancies | | | | 1 | | 2 | | 8 |  |
|  | 1. Prolonged labour | | | | 1 | | 2 | | 8 |  |
|  | 1. Obstructed labour | | | |  | |  | |  |  |
|  | 1. C- section | | | | 1 | | 2 | | 8 |  |
|  | 1. Assisted delivery (forceps, vacuum extraction) | | | | 1 | | 2 | | 8 |  |
|  | 1. Prior neonatal death (death of baby < 1 month) | | | | 1 | | 2 | | 8 |  |
|  | 1. Prior stillbirth | | | | 1 | | 2 | | 8 |  |
|  | 1. Prior abortion/ miscarriage | | | | 1 | | 2 | | 8 |  |
| **Examination of the pregnant woman** | | | | | | | | | | |
| 111 | Washes hands **appropriately (with soap & water or using alcohol hand rub)** | | | | | | | | |  |
|  | a) **BEFORE** general examination | | | | 1 | | 2 | | 8 |  |
|  | b) **AFTER** general examination | | | | 1 | | 2 | | 8 |  |
| 112 | Explains procedures before proceeding | | | | 1 | | 2 | | 8 |  |
| 113 | **Performs the following steps for general examination** | | | | | | | | |  |
|  | 1. Takes temperature by thermometer | | | | 1 | | 2 | | 8 |  |
|  | 1. Counts pulse | | | | 1 | | 2 | | 8 |  |
|  | 1. Takes blood pressure | | | | 1 | | 2 | | 8 |  |
|  | 1. Edema checked (pedal edema) | | | | 1 | | 2 | | 8 |  |
|  | 1. Anemia checked by checking eye/ tongue/ palm | | | | 1 | | 2 | | 8 |  |
| 114 | Asks/notes amount, colour of urine | | | | 1 | | 2 | | 8 |  |
| 115 | Blood sample taken | | | | 1 | | 2 | | 8 |  |
| 116 | Urine tested for presence of protein | | | | 1 | | 2 | | 8 |  |
| 117 | IV line was set on woman | | | | 1 | | 2 | | 8 |  |
| 118 | **Abdominal examination** was performed | | | | 1 | | 2 | |  | No🡪 120 |
| 119 | a) Checks fundal height with measuring tape | | | | 1 | | 2 | | 8 |  |
|  | b) Checks fetal presentation by palpation of abdomen | | | | 1 | | 2 | | 8 |  |
|  | c) Checks fetal heart rate with stethoscope/Doppler | | | | 1 | | 2 | | 8 |  |
| 120 | **Vaginal examination** was performed | | | | 1 | | 2 | |  | No🡪126 |
| 121 | Washes hands appropriately **BEFORE** examination | | | | 1 | 2 | | | 8 |  |
| 122 | Wears sterile gloves for vaginal examination | | | | 1 | 2 | | | 8 |  |
| 123 | Informs woman about procedure **BEFORE** examination | | | | 1 | 2 | | | 8 |  |
| 124 | Informs the woman about findings **AFTER** examination | | | | 1 | 2 | | | 8 |  |
| 125 | Washes hands appropriately **AFTER** examination | | | | 1 | 2 | | | 8 |  |
| 126 | Plan for delivery is discussed with the woman  (NVD or Assisted vaginal delivery or CS) | | | | 1 | 2 | | | 8 |  |
| 127 | Was this woman referred for a C- section | | | | 1 | 2 | | |  | No🡪129 |
| 128 | Cause of referral (multiple answer is possible) | | Obstructed labor | | | | | | A |  |
|  |  |  | Pre- eclampsia/ Eclampsia | | | | | | B |  |
|  |  |  | Placental praevia | | | | | | C |  |
|  |  |  | Previous c- section scar | | | | | | D |  |
|  |  |  | Fetal distress | | | | | | E |  |
|  |  |  | Cord prolapsed | | | | | | F |  |
|  |  |  | Maternal distress | | | | | | G |  |
|  |  |  | Prolonged labor | | | | | | H |  |
|  |  |  | Malposition (breech) | | | | | | I |  |
|  |  |  | Other (specify): _________________________ | | | | | | Y |  |
| 129 | Did the health worker start a partograph? | | | | 1 | 2 | | | 8 |  |
| *Now based on your opinion circle the* ***best choice*** *for* ***QUESTION 130-133*** | | | | | | | | | | |
| 130 | How was the initial reception of the health worker (HW) to woman | | | Always welcoming | | | | | 1 |  |
|  |  |  |  | Sometimes welcoming | | | | | 2 |  |
|  |  |  |  | Often unfriendly | | | | | 3 |  |
| 131 | How was overall communication between HW and woman | | | Courteous | | | | | 1 |  |
|  |  |  |  | Rushed | | | | | 2 |  |
|  |  |  |  | Harsh | | | | | 3 |  |
| 132 | How did woman feel talking to the health worker? | | | Happy | | | | | 1 |  |
|  |  |  |  | Indifferent | | | | | 2 |  |
|  |  |  |  | Timid/scared | | | | | 3 |  |
| 133 | Was woman‘s privacy ensured/maintained during interaction with the HW? | | | Always | | | | | 1 |  |
|  |  |  |  | Sometimes | | | | | 2 |  |
|  |  |  |  | Never | | | | | 3 |  |
| **End of section 1; Please go to section 2** | | | | | | | | | | |

**Section 2: Intermittent Observation of First Stage of Labor**

| **No** | **Questions and filters** | | | | | | | | | **Options and coding** | | | | | | **Skip** |
| --- | --- | --- | --- | --- | --- | --- | --- | --- | --- | --- | --- | --- | --- | --- | --- | --- |
|  |  |  |  |  |  |  |  |  |  | **Yes** | | **No** | | | |  |
| 200 | Was this section observed? | | | | | | | | | 1 | | 2 | | | | Yes 🡪202 |
| 201 | *If No, mention the reason* |  | | | | | | | | | | | | | | Skip to  **Section 3** |
| **General observation of labour room or ward or area** | | | | | | | | | | | | | | | | |
| 202 | How is the layout of the labour ward? | | | | | | Separate room for each patient | | | | | | | | 1 |  |
|  |  |  |  |  |  |  | Each patient's bed is partitioned | | | | | | | | 2 |  |
|  |  |  |  |  |  |  | Open with no privacy | | | | | | | | 3 |  |
| 203 | How would you describe the patient load in the labour room? | | | | | | All beds filled and some on the floor | | | | | | | | 1 |  |
|  |  |  |  |  |  |  | All beds filled but nobody on floor | | | | | | | | 2 |  |
|  |  |  |  |  |  |  | Some beds are filled, not all | | | | | | | | 3 |  |
|  |  |  |  |  |  |  | Almost empty | | | | | | | | 4 |  |
| 204 | How many beds are in the labour ward? | | | | | | | | | | |  | | | |  |
| 205 | How many staff are on duty? | | | | | | | | | | |  | | | |  |
| 206 | Clean surface / bed for delivery available? | | | | | | | | 1 | | | 2 | | | |  |
| 207 | Light and ventilation in the room adequate? | | | | | | | | 1 | | | 2 | | | |  |
| *Record whether the provider carried out the following steps and/or examinations:*  *(some of the following steps may be performed simultaneously or by more than one provider)* | | | | | | | | | | | | | | | | |
| **Progress of Labour** | | | | | | | | | | | | | | | | |
| **No** | **Questions and filters** | | | | | | | | | **Options and coding** | | | | | | **Skip** |
|  |  |  |  |  |  |  |  |  |  | **Yes** | **No** | | | **DK** | |  |
| 208 | Explains what will happen during labour | | | | | | | | | 1 | 2 | | | 8 | |  |
| 209 | Encourages to consume fluid/food during labour | | | | | | | | | 1 | 2 | | | 8 | |  |
| 210 | Encourages/assists woman to ambulate, adopt different position during labour | | | | | | | | | 1 | 2 | | | 8 | |  |
| 211 | Plan for delivery discussed with mother | | | | | | | | | 1 | 2 | | | 8 | |  |
| 212 | Is a support person present at any point during labor? | | | | | | | | | 1 | 2 | | | 8 | |  |
| 213 | Partograph started to monitor progress of labour | | | | | | | | | 1 | 2 | | |  | | No🡪220 |
| 214 | Action line plotted | | | | | | | | | 1 | 2 | | |  | | No🡪220 |
| 215 | Action line on partograph reached | | | | | | | | | 1 | 2 | | |  | | No🡪220 |
| 216 | *Record time, when action line was reached in partograph (Record in 24 hours)* | | | | | | | | | **:**  **h h m m** | | | | | |  |
| 217 | Action line reached🡪Any definitive action taken? | | | | | | | | | 1 | 2 | | | 8 | | No/ DK🡪220 |
| 218 | *Record time, when definitive action was taken*  *(Record in 24 hours)* | | | | | | | | | **:**  **h h m m** | | | | | |  |
| 219 | What definitive action was taken: | | | | Consulted with senior doctor of same facility | | | | | | | | | | A |  |
|  |  |  |  |  | Referred to other facility | | | | | | | | | | B |  |
|  |  |  |  |  | Prepared for Assisted delivery | | | | | | | | | | C |  |
|  |  |  |  |  | Prepared for C-section | | | | | | | | | | D |  |
|  |  |  |  |  | Others (specify) _______________________ | | | | | | | | | | Y |  |
| **Examination & Procedures** | | | | | | | | | | | | | | | | |
| **No** | **Questions and filters** | | | | | | | | | **Options and coding** | | | | | | **Skip** |
|  |  |  |  |  |  |  |  |  |  | **Yes** | **No** | | | **DK** | |  |
| 220 | Puts on clean protective clothing in preparation for birth (mackintosh, goggles, gown or apron) | | | | | | | | | 1 | 2 | | | 8 | |  |
| 221 | Drapes woman (1 under buttocks, 1 over abdomen) | | | | | | | | | 1 | 2 | | | 8 | |  |
| 222 | Woman was examined in the labour ward | | | | | | | | | 1 | 2 | | | 8 | | No/DK🡪231 |
| 223 | Wash hand appropriately **BEFORE** any examination | | | | | | | | | 1 | 2 | | | 8 | |  |
| 224 | Wears sterile surgical gloves | | | | | | | | | 1 | 2 | | | 8 | |  |
| 225 | Explains procedures before proceeding | | | | | | | | | 1 | 2 | | | 8 | |  |
| 226 | How often was she examined in the labor ward? | | | | | | | Half-hourly | | | | | | 1 | |  |
|  |  |  |  |  |  |  |  | Hourly | | | | | | 2 | |  |
|  |  |  |  |  |  |  |  | 2-4hourly | | | | | | 3 | |  |
|  |  |  |  |  |  |  |  | more than 4 hourly | | | | | | 4 | |  |
| 227 | How often were partographs filled after examination? | | | | | | | Never | | | | | | 1 | |  |
|  |  |  |  |  |  |  |  | Sometimes | | | | | | 2 | |  |
|  |  |  |  |  |  |  |  | After each examination | | | | | | 3 | |  |
| 228 | Vaginal examination done at each examination? | | | | | | | | | 1 | 2 | | | 8 | |  |
| 229 | Privacy maintained during examination? | | | | | | | | | 1 | 2 | | | 8 | |  |
| 230 | Who did the examination?  *(Circle the highest ranked)* | | Doctor | | | | | | | | | | | 1 | |  |
|  |  |  | Midwife | | | | | | | | | | | 2 | |  |
|  |  |  | Nurse | | | | | | | | | | | 3 | |  |
|  |  |  | Untrained nurse (no EOC/midwifery training) | | | | | | | | | | | 4 | |  |
| 231 | Augments labor with oxytocin | | | | | | | | | 1 | 2 | | | 8 | | No/ DK🡪 233 |
| 232 | Oxytocin administered intravenously (IV) | | | | | | | | | 1 | 2 | | | 8 | |  |
| 233 | Performs artificial rupture of membrane | | | | | | | | | 1 | 2 | | | 8 | |  |
| 234 | Administers antibiotics | | | | | | | | | 1 | 2 | | | 8 | | No/ DK🡪237 |
| 235 | Why were antibiotics administered? | | | | | Treatment for chorio-amnionitis | | | | | | | | 1 | |  |
|  |  |  |  |  |  | Management of pre-labor rupture of membranes | | | | | | | | 2 | |  |
|  |  |  |  |  |  | Preparation for C-section | | | | | | | | 3 | |  |
|  |  |  |  |  |  | Routine/prophylactic | | | | | | | | 4 | |  |
|  |  |  |  |  |  | Others (specify) _________________________________ | | | | | | | | 7 | |  |
|  |  |  |  |  |  | Don’t know | | | | | | | | 8 | |  |
| 236 | Which antibiotic was administered? *(Circle all that apply)* | | | | | Penicillin | | | | | | | | A | |  |
|  |  |  |  |  |  | Ampicillin | | | | | | | | B | |  |
|  |  |  |  |  |  | Gentamicin | | | | | | | | C | |  |
|  |  |  |  |  |  | Metronidazole | | | | | | | | D | |  |
|  |  |  |  |  |  | Cephalosporin | | | | | | | | E | |  |
|  |  |  |  |  |  | Other ______________________ | | | | | | | | Y | |  |
|  |  |  |  |  |  | Don’t know | | | | | | | | Z | |  |
| 237 | Attitude of health workers when woman is in pain | | | | | Caring & supportive | | | | | | | | 1 | |  |
|  |  |  |  |  |  | Indifferent | | | | | | | | 2 | |  |
|  |  |  |  |  |  | Abusive (verbal and physical) | | | | | | | | 3 | |  |
| 238 | Did woman request for anything and not given? | | | | | | | | | 1 | 2 | | | 8 | | No/ DK🡪 240 |
| 239 | Was woman told respectfully why request denied? | | | | | | | | | 1 | 2 | | | 8 | |  |
| 240 | Did woman have an IV line access? | | | | | | | | | 1 | 2 | | | 8 | |  |
| 241 | Was this woman referred for a C- section | | | | | | | | | 1 | 2 | | |  | | No🡪243 |
| 242 | Cause of referral (multiple answer is possible) | | | Obstructed labor | | | | | | | | | | A | |  |
|  |  |  |  | Pre- eclampsia/ Eclampsia | | | | | | | | | | B | |  |
|  |  |  |  | Placental praevia | | | | | | | | | | C | |  |
|  |  |  |  | Previous c- section scar | | | | | | | | | | D | |  |
|  |  |  |  | Fetal distress | | | | | | | | | | E | |  |
|  |  |  |  | Cord prolapsed | | | | | | | | | | F | |  |
|  |  |  |  | Maternal distress | | | | | | | | | | G | |  |
|  |  |  |  | Prolonged labor | | | | | | | | | | H | |  |
|  |  |  |  | Malposition (breech) | | | | | | | | | | I | |  |
|  |  |  |  | Other (specify): _______________________ | | | | | | | | | | Y | |  |
| 243 | Has the woman completed the first stage of labor? | | | | | | | | | 1 | 2 | |  | | | Yes🡪Section 3 |
| **End of Section 2; Please go to section 3** | | | | | | | | | | | | | | | | |

**Section 3: Continuous Observation of Second & Third Stage of Labor**

| **No** | **Questions and filters** | | | | | | | | | **Options and coding** | | | | | | | | | **Skip** |
| --- | --- | --- | --- | --- | --- | --- | --- | --- | --- | --- | --- | --- | --- | --- | --- | --- | --- | --- | --- |
|  |  |  |  |  |  |  |  |  |  | **Yes** | | | | **No** | | | | |  |
| 300 | Was this section observed? | | | | | | | | | 1 | | | | 2 | | | | | Yes🡪302 |
| 301 | *If No, mention the reason* |  | | | | | | | | | | | | | | | | | Skip to  **Section 4** |
| *Record whether the provider carried out the following steps and/or examinations:*  *(some of the following steps may be performed simultaneously or by more than one provider)* | | | | | | | | | | | | | | | | | | | |
| **Preparation at Delivery ward or room or area** | | | | | | | | | | | | | | | | | | | |
| *Observe the area and circle the appropriate answer for following items if available* | | | | | | | | | | | | | | | | | | | |
| 302 | How is the layout of the delivery area? | | Separate room for each client | | | | | | | | | | | | | | 1 | |  |
|  |  |  | Separate bed for each client; screened partition | | | | | | | | | | | | | | 2 | |  |
|  |  |  | Many patients to a room, no privacy | | | | | | | | | | | | | | 3 | |  |
| 303 | How is cleanliness of delivery area? | | Clean | | | | | | | | | | | | | | 1 | |  |
|  |  |  | Unclean | | | | | | | | | | | | | | 2 | |  |
| 304 | Delivery bed | | | | | | | | | 1 | | | | 2 | | | | |  |
| 305 | Timer (clock or watch with seconds hand) | | | | | | | | | 1 | | | | 2 | | | | |  |
| 306 | Wall thermometer | | | | | | | | | 1 | | | | 2 | | | | |  |
| 307 | Wooden box/stair case beside the delivery bed | | | | | | | | | 1 | | | | 2 | | | | |  |
| 308 | OT Light | | | | | | | | | 1 | | | | 2 | | | | |  |
| 309 | Weight machine for baby | | | | | | | | | 1 | | | | 2 | | | | |  |
| 310 | Sterile gloves | | | | | | | | | 1 | | | | 2 | | | | |  |
| 311 | Catheter for woman | | | | | | | | | 1 | | | | 2 | | | | |  |
| 312 | Two cloths/blankets (1 for drying, 1 for wrapping) | | | | | | | | | 1 | | | | 2 | | | | |  |
| 313 | Cap/hat for the newborn | | | | | | | | | 1 | | | | 2 | | | | |  |
| 314 | Is there a delivery tray? | | | | | | | | | 1 | | | | 2 | | | | | No 🡪 316 |
| 315 | List the contents of the delivery tray (circle all that applied) | | | | | | | | | | | | | | | | | |  |
|  | 1. Suture and needle | | | | | | | | | 1 | | | | 2 | | | | |  |
|  | 1. Cord clamp | | | | | | | | | 1 | | | | 2 | | | | |  |
|  | 1. Clean sterile gauze pack | | | | | | | | | 1 | | | | 2 | | | | |  |
|  | 1. Sharp scissors | | | | | | | | | 1 | | | | 2 | | | | |  |
|  | 1. Oxytocin | | | | | | | | | 1 | | | | 2 | | | | |  |
| 316 | Is there a newborn resuscitation area? | | | | | | | | | 1 | | | | 2 | | | | | No 🡪 318 |
| 317 | List the items in the resuscitation area (circle all that applied) | | | | | | | | | | | | | | | | | |  |
|  | 1. Ambu bag | | | | | | | | | 1 | | | | 2 | | | | |  |
|  | 1. Self-inflating ventilation bag (250 or 500 mL) | | | | | | | | | 1 | | | | 2 | | | | |  |
|  | 1. Newborn face mask size 0 | | | | | | | | | 1 | | | | 2 | | | | |  |
|  | 1. Newborn face mask size 1 | | | | | | | | | 1 | | | | 2 | | | | |  |
|  | 1. Suction bulb/ penguin sucker | | | | | | | | | 1 | | | | 2 | | | | |  |
|  | 1. Suction machine | | | | | | | | | 1 | | | | 2 | | | | |  |
|  | 1. Radiant warmer | | | | | | | | | 1 | | | | 2 | | | | |  |
|  | 1. Bulb syringe for aspiration of fluids | | | | | | | | | 1 | | | | 2 | | | | |  |
|  | 1. Oxygen cylinder with oxygen | | | | | | | | | 1 | | | | 2 | | | | |  |
| **Preparation for Delivery** | | | | | | | | | | | | | | | | | | | |
| 318 | Puts on clean protective clothing in preparation for birth (mackintosh, goggles, gown or apron) | | | | | | | | | 1 | | | 2 | | | | 8 | |  |
| 319 | Washes hands appropriately before any examination | | | | | | | | | 1 | | | 2 | | | | 8 | | No/DK🡪 321 |
| 320 | Method of drying hands | | | | | | With clean regular towels | | | | | | | | | | 1 | |  |
|  |  |  |  |  |  |  | Disposable towels | | | | | | | | | | 2 | |  |
|  |  |  |  |  |  |  | Air dry | | | | | | | | | | 3 | |  |
|  |  |  |  |  |  |  | Didn’t dry hands | | | | | | | | | | 4 | |  |
|  |  |  |  |  |  |  | Others (specify) __________ | | | | | | | | | | 7 | |  |
| 321 | Checks delivery trolley/instrument for functioning status | | | | | | | | | 1 | | | 2 | | | | 8 | |  |
| 322 | Checks resuscitation equipment for functioning status | | | | | | | | | 1 | | | 2 | | | | 8 | |  |
| 323 | Drapes woman appropriately for delivery | | | | | | | | | 1 | | | 2 | | | | 8 | |  |
| 324 | Wears sterile surgical gloves *(yes if no contamination)* | | | | | | | | | 1 | | | 2 | | | | 8 | |  |
| 325 | Puts on two pairs of sterile gloves on both hands | | | | | | | | | 1 | | | 2 | | | | 8 | |  |
| 326 | Woman asked for her preferred delivery position | | | | | | | | | 1 | | | 2 | | | | 8 | |  |
| 327 | Clean vulva/perineum with antiseptic solution | | | | | | | | | 1 | | | 2 | | | | 8 | |  |
| 328 | Epidural given for the delivery | | | | | | | | | 1 | | | 2 | | | | 8 | |  |
| 329 | Performs episiotomy | | | | | | | | | 1 | | | 2 | | | |  | | No🡪331 |
| 330 | Mentions to mother why episiotomy is performed | | | | | | | | | 1 | | | 2 | | | |  | |  |
| 331 | Presentation of baby | Cephalic (head first) | | | | | | | | | | | | | | | 1 | |  |
|  |  | Limb first | | | | | | | | | | | | | | | 2 | |  |
|  |  | Buttock first | | | | | | | | | | | | | | | 3 | |  |
|  |  | Others (specify)___________________________ | | | | | | | | | | | | | | | 7 | |  |
| **Delivery & Uterotonic** | | | | | | | | | | | | | | | | | | | |
| 332 | Who conducted the delivery? | Doctor | | | | | | | | | | | | | | | 1 | |  |
|  |  | Midwife | | | | | | | | | | | | | | | 2 | |  |
|  |  | Nurse | | | | | | | | | | | | | | | 3 | |  |
|  |  | Untrained nurse(no EOC/ no midwifery training) | | | | | | | | | | | | | | | 4 | |  |
|  |  | Others (specify)_________________________ | | | | | | | | | | | | | | | 7 | |  |
| 333 | Supports perineum as baby's head is delivered | | | | | | | | | 1 | | | 2 | | | | 8 | |  |
| 334 | *Record time of the delivery of the baby*  *(Record in 24 hour)* | | | | | | | | | **:**  h h m m | | | | | | | | |  |
| 335 | Checks for another baby prior to giving the uterotonic | | | | | | | | | 1 | | 2 | | | 8 | | | |  |
| 336 | 2^nd^ baby present? *(observer: circle 1 if multiple babies)* | | | | | | | | | 1 | | 2 | | |  | | | |  |
| 337 | Administers uterotonic (oxytocin)? | | | | | | | | | 1 | | 2 | | |  | | | | No🡪344 |
| 338 | *Record time when uterotonic is given*  *(Record in 24 hour)* | | | | | | | | | **:**  h h m m | | | | | | | | |  |
| 339 | Timing of administration of uterotonic | | | | At delivery of anterior shoulder | | | | | | | | | | | 1 | | |  |
|  |  |  |  |  | Within 1 min of delivery of baby | | | | | | | | | | | 2 | | |  |
|  |  |  |  |  | Within 3 min of delivery of baby | | | | | | | | | | | 3 | | |  |
|  |  |  |  |  | More than 3 min after delivery of baby | | | | | | | | | | | 4 | | |  |
| 340 | Which uterotonic given  *(multiple answers possible)* | | | | Oxytocin | | | | | | | | | | | A | | |  |
|  |  |  |  |  | Ergometrine | | | | | | | | | | | B | | |  |
|  |  |  |  |  | Syntometrine | | | | | | | | | | | C | | |  |
|  |  |  |  |  | Misoprostol | | | | | | | | | | | D | | |  |
| 341 | *Record dose of uterotonic given (ask if necessary)* | | | | **Uterotonic 1** | | | | | **Uterotonic 2** | | | | | | | | |  |
|  |  |  |  |  |  | | | | |  | | | | | | | | |  |
| 342 | Units of medication *(observer: if necessary, ask afterwards)* | | | | IU | | | | A | IU | | | | | | | | A |  |
|  |  |  |  |  | Mg | | | | B | Mg | | | | | | | | B |  |
|  |  |  |  |  | mL | | | | C | mL | | | | | | | | C |  |
|  |  |  |  |  | Mcg | | | | D | Mcg | | | | | | | | D |  |
| 343 | Route uterotonic given: | | | | IM | | | | A | IM | | | | | | | | A |  |
|  |  |  |  |  | IV | | | | B | IV | | | | | | | | B |  |
|  |  |  |  |  | Oral | | | | C | Oral | | | | | | | | C |  |
|  |  |  |  |  | Per rectal | | | | D | Per rectal | | | | | | | | D |  |
| 344 | *Record time the cord was clamped*  *(Record in 24 hour)* | | | | | | | | | **:**  h h m m | | | | | | | | |  |
| 345 | Applies traction to cord & supra-pubic counter traction | | | | | | | | | 1 | | 2 | | | 8 | | | |  |
| 346 | Uterine massage immediately after placenta delivery | | | | | | | | | 1 | | 2 | | | 8 | | | |  |
| 347 | *Record time when placenta was delivered (24 hour)* | | | | | | | | | **:**  h h m m | | | | | | | | |  |
| 348 | Assesses completeness of placenta and membranes | | | | | | | | | 1 | | 2 | | | 8 | | | |  |
| 349 | Assesses for perineal and vaginal laceration | | | | | | | | | 1 | | 2 | | | 8 | | | |  |
| 350 | *Observer: Did more than one HW assist with the birth?* | | | | | | | | | 1 | | 2 | | |  | | | |  |
| 351 | *Observer: Did mother gave birth in lithotomy position?* | | | | | | | | | 1 | | 2 | | |  | | | |  |
| 352 | *Observer: Is a support person present at birth?* | | | | | | | | | 1 | | 2 | | |  | | | | No🡪 354 |
| 353 | Who was present? | | | | | | | Husband | | | | | | | 1 | | | |  |
|  |  |  |  |  |  |  |  | Mother/ Mother in law | | | | | | | 2 | | | |  |
|  |  |  |  |  |  |  |  | Other relative/ friend | | | | | | | 3 | | | |  |
|  |  |  |  |  |  |  |  | Neighbour | | | | | | | 4 | | | |  |
|  |  |  |  |  |  |  |  | Other (specify)__________ | | | | | | | 7 | | | |  |
| 354 | Did woman request for support person during delivery? | | | | | | | | | 1 | | 2 | | | 8 | | | |  |
| 355 | Was there any complication during labour and delivery? | | | | | | | | | 1 | | 2 | | | 8 | | | | No🡪 357 |
| 356 | What complications?  *(Multiple answers possible)* | | | Post-partum haemorrhage | | | | | | | | | | | A | | | |  |
|  |  |  |  | Perineal tear | | | | | | | | | | | B | | | |  |
|  |  |  |  | Obstetric Fistula | | | | | | | | | | | C | | | |  |
|  |  |  |  | Ruptured uterus | | | | | | | | | | | D | | | |  |
|  |  |  |  | Eclampsia/Pre-eclampsia | | | | | | | | | | | E | | | |  |
|  |  |  |  | Obstructed labour | | | | | | | | | | | F | | | |  |
|  |  |  |  | Prolong labour | | | | | | | | | | | G | | | |  |
|  |  |  |  | Others (specify)___________ | | | | | | | | | | | Y | | | |  |
| 357 | Woman examined by a HW after the delivery | | | | | Within 15 minutes | | | | | | | | | 1 | | | |  |
|  |  |  |  |  |  | Within 30 minutes | | | | | | | | | 2 | | | |  |
|  |  |  |  |  |  | Not examined | | | | | | | | | 3 | | | |  |
| 358 | Record time, when labour & delivery observation ends  *(Record in 24 hour)* | | | | | | | | | **:**h h m m | | | | | | | | |  |
| 359 | *Observer:* Did you see any HW filling out partograph after delivery with information that was supposed to be plotted during labour*? (circle “8” if partograph was not initiated)* | | | | | | | | | 1 | 2 | | | | | | 8 | |  |
| **End of section 3; Please go to section 4** | | | | | | | | | | | | | | | | | | | |

**Section 4: Immediate Newborn and Post-partum Care**

| **No** | **Questions and filters** | | | | **Options and coding** | | | | **Skip** |
| --- | --- | --- | --- | --- | --- | --- | --- | --- | --- |
|  |  |  |  |  | **Yes** | | **No** | |  |
| 400 | Was this section observed? | | | | 1 | | 2 | | Yes 🡪 402 |
| 401 | If No, mention the reason |  | | | | | | | Skip to  Section 5 |
| *Record whether the provider carried out the following steps and/or examinations:*  *(some of the following steps may be performed simultaneously or by more than one provider)* | | | | | | | | | |
| **Immediate newborn care** | | | | | | | | | |
| **No** | **Questions and filters** | | | | **Options and coding** | | | | **Skip** |
|  |  |  |  |  | **Yes** | **No** | | **DK** |  |
| 402 | Is there a separate staff member who will be taking care of the newborn? | | | | 1 | 2 | | 8 |  |
| 403 | Who took care of the newborn? | | | Doctor | | | | 1 |  |
|  |  |  |  | Midwife | | | | 2 |  |
|  |  |  |  | Nurse | | | | 3 |  |
|  |  |  |  | Other (specify) ____________ | | | | 7 |  |
| 404 | *Record time of delivery*  *(Record in 24 hour)* | | | | **:**  h h m m | | | |  |
| 405 | Birth attendant notes/shouts time of delivery | | | | 1 | 2 | | 8 |  |
| 406 | Baby delivered on mother's abdomen | | | | 1 | 2 | | 8 |  |
| 407 | Immediately dries baby with towel thoroughly | | | | 1 | 2 | | 8 |  |
| 408 | Discards the wet towel | | | | 1 | 2 | | 8 |  |
| 409 | Spontaneous breathing assessed at birth | | | | 1 | 2 | | 8 |  |
| 410 | Is the baby breathing or crying | | | | 1 | 2 | |  |  |
| 411 | Places baby on mother’s abdomen “skin to skin” | | | | 1 | 2 | | 8 | No/ DK🡪413 |
| 412 | *Record the time of placing on mother’s abdomen “skin to skin” (Record in 24 hour)* | | | | **:**  h h m m | | | |  |
| 413 | If not placed skin to skin, wraps baby in dry towel | | | | 1 | 2 | | 8 |  |
| 414 | Cord was touched with fresh pair of gloves worn | | | | 1 | 2 | | 8 |  |
| 415 | Ties or clamps cord after 1 minute | | | | 1 | 2 | | 8 | No/ DK🡪419 |
| 416 | Cuts cord with sterile blade or sterile scissors | | | | 1 | 2 | | 8 | No/ DK🡪419 |
| 417 | *Record the time (cord cutting)*  *(Record in 24 hour)* | | | | **:**  h h m m | | | |  |
| 418 | Placenta & cord removed by cont. cord traction | | | | 1 | 2 | | 8 |  |
| 419 | Chlorhexidine applied to the cord | | | | 1 | 2 | | 8 |  |
| 420 | Any other thing applied to the cord | | | | 1 | 2 | | 8 | No/DK🡪422 |
| 421 | What was applied (specify) | | | |  | | | |  |
| 422 | Is a support person for mother present? | | | | 1 | 2 | |  |  |
| **Health Check** | | | | | | | | | |
| 423 | Checks baby's temperature 15 minutes after birth | | | | 1 | 2 | | 8 | No/DK🡪 425 |
| 424 | *Record Baby's temperature*  *(record in degree Celsius)* | | | | . | | | |  |
| 425 | Checks baby's skin colour 15 minutes after birth | | | | 1 | 2 | | 8 |  |
| 426 | Checks mother's BP within 30 minutes after birth | | | | 1 | 2 | | 8 |  |
| 427 | Palpates uterus 15 min. after delivery of placenta | | | | 1 | 2 | | 8 |  |
| **First Hour After Birth** | | | | | | | | | |
| 428 | Mother & newborn kept in same room | | | | 1 | 2 | | 8 |  |
| 429 | Baby bathed within the first hour after birth | | | | 1 | 2 | | 8 |  |
| 431 | Baby was kept “skin to skin” continuously during first hour after birth | | | | 1 | 2 | | 8 | Yes/DK 🡪433 |
| 432 | *Record the time baby first taken out of skin to skin contact with mother (Record in 24 hour)* | | | | **:**  h h m m | | | |  |
| 433 | Was breastfeeding initiated within the first hour after birth | | | | 1 | 2 | | 8 |  |
| 434 | *Record the time when baby was first put to the breast (Record in 24 hour)* | | | | **:**  h h m m | | | |  |
| 435 | Attachment for breast feeding was correct | | | | 1 | 2 | | 8 |  |
| 436 | Baby suckled properly | | | | 1 | 2 | | 8 |  |
| 437 | Baby was weighed by neonatal weighing scale | | | | 1 | 2 | | 8 | No/ DK🡪440 |
| 438 | *Record weight of the baby*  *(Record in kg)* | | | | . | | | |  |
| 440 | Thorough physical examination of baby done | | | | 1 | 2 | | 8 |  |
| 441 | Provides tetracycline eye ointment 1% | | | | 1 | 2 | | 8 |  |
| 442 | Administers Vitamin K to newborn | | | | 1 | 2 | | 8 |  |
| 443 | Administers post-partum antibiotics to mother | | | | 1 | 2 | | 8 | Yes 🡪skip 444 & 445 |
| 444 | Why were antibiotics administered? | | Treatment for chorio-amnionitis | | | | | 1 | |
|  |  |  | Routine/prophylactic | | | | | 2 | |
|  |  |  | Third stage/postpartum procedure | | | | | 3 | |
|  |  |  | Don’t know | | | | | 8 | |
|  |  |  | Others (specify)__________________ | | | | | 7 | |
| 445 | Which antibiotic was administered? | | Penicillin | | | | | A | |
|  |  |  | Ampicillin | | | | | B | |
|  |  |  | Gentamicin | | | | | C | |
|  |  |  | Metronidazole | | | | | D | |
|  |  |  | Cephalosporin | | | | | E | |
|  |  |  | Others (specify)  _________________________ | | | | | Y | |
|  |  |  | Don’t know | | | | | Z | |
| **Remember to thank client and health worker for their participation in the study** | | | | | | | | | |
| **End of section 4; Please go to section 6** | | | | | | | | | |

**Section 5: Checklist for Newborn Resuscitation**

| **No** | **Questions and filters** | | **Options and coding** | | | | | **Skip** |
| --- | --- | --- | --- | --- | --- | --- | --- | --- |
|  |  |  | **Yes** | | | **No** | |  |
| 500 | Was this section observed? | | 1 | | | 2 | | Yes🡪502 |
| 501 | *If No, mention the reason* |  | | | | | | Skip to Section 6 |
| *Record whether the provider carried out the following steps and/or examinations:*  *(some of the following steps may be performed simultaneously or by more than one provider)* | | | | | | | | |
| **Immediate care** | | | | | | | | |
| **No** | **Questions and filters** | | **Options and coding** | | | | | **Skip** |
|  |  |  | **Yes** | **No** | | | **DK** |  |
| 502 | *Record time, when resuscitation started*  *(Record in 24 hour)* | | **:**  h h m m | | | | |  |
| 503 | Clears airway by suctioning mouth first, then nose | | 1 | 2 | | | 8 |  |
| 504 | 1. Stimulates baby by back rubbing | | 1 | 2 | | | 8 |  |
| 505 | *Does newborn starts to breathe or cry?* | | 1 | 2 | | |  | Yes🡪 536 |
| 506 | HW calls for help | | 1 | 2 | | | 8 |  |
| 507 | Tells woman (and support person) what is going to happen | | 1 | 2 | | | 8 |  |
| 508 | Listens to woman, provides support and assurance | | 1 | 2 | | | 8 |  |
| 509 | Ties or clamps cord immediately | | 1 | 2 | | | 8 |  |
| 510 | Cuts cord with sterile blade or sterile scissors | | 1 | 2 | | | 8 |  |
| 511 | Places baby on its back on clean, warm surface or towel | | 1 | 2 | | | 8 |  |
| 512 | Places head in slightly extended position to open airway | | 1 | 2 | | | 8 |  |
| 513 | Checks mouth, throat, nose for secretions, clears if needed | | 1 | 2 | | | 8 |  |
| 514 | Places correct-sized mask over baby’s face so that it covers the chin, mouth and nose (but not eyes) | | 1 | 2 | | | 8 |  |
| 515 | *Record the time of putting the mask on*  *(Record in 24 hour)* | | **:**  h h m m | | | | |  |
| 516 | Checks seal by ventilating twice and observing rise of chest | | 1 | 2 | | | 8 |  |
| 517 | *Observe: Is baby’s chest rising in response to ventilation?* | | 1 | 2 | | |  | Yes🡪 526 |
| 518 | Checks position of baby’s head to make sure slightly extended position of neck (not blocking airway) | | 1 | 2 | | | 8 |  |
| 519 | Checks mouth, throat, nose for secretions, clears if needed | | 1 | 2 | | | 8 |  |
| 520 | Checks seal by ventilating twice and observing rise of chest | | 1 | 2 | | | 8 |  |
| 521 | *Observe: Is baby’s chest rising in response to ventilation?* | | 1 | 2 | | |  | Yes🡪 526 |
| 522 | Again, checks position of baby’s head to make sure slightly extended position of neck | | 1 | 2 | | | 8 |  |
| 523 | Repeats suction of mouth, nose to clear secretions | | 1 | 2 | | | 8 |  |
| 524 | Checks seal by ventilating twice and observing rise of chest | | 1 | 2 | | | 8 |  |
| 525 | *Observe: Is baby’s chest rising in response to ventilation?* | | 1 | 2 | | |  | No🡪 533 |
| 526 | Ventilates at a rate of 30 to 50 breaths/minute | | 1 | 2 | | | 8 |  |
| 527 | Conducts breathing assessment 1 minute after ventilation | | 1 | 2 | | |  | No🡪 529 |
| 528 | Condition of newborn at assessment | | | | | | **Code** |  |
|  | Respiration rate 30-50 breaths/minute and no chest in-drawing | | | | | | 1 | 🡪 533 |
|  | Respiration rate <30 breaths/minute with severe in-drawing | | | | | | 2 | 🡪 529 |
|  | No spontaneous breathing | | | | | | 3 |  |
| 529 | Continues prolonged ventilation (for 10 minutes) | | 1 | 2 | | | 8 |  |
| 530 | Conducts breathing assessment after prolonged ventilation | | 1 | 2 | | |  | No🡪 533 |
| 531 | Condition of newborn at assessment | | | | | | **Code** |  |
|  | Respiration rate 30-50 breaths/minute and no chest in-drawing | | | | | | 1 | 🡪 533 |
|  | Respiration rate <30 breaths/minute with severe in-drawing | | | | | | 2 |  |
|  | No spontaneous breathing | | | | | | 3 |  |
| 532 | Continues Ventilation | | 1 | 2 | | | 8 |  |
| 533 | *Record time that resuscitation actions ended* (or time of death if baby died) | | **:**  h h m m | | | | |  |
| 534 | Was the resuscitation successful? | | 1 | | 2 | |  | yes 🡪536 |
| 535 | What was done if resuscitation not succeeded? | Arranges transfer to special care/NICU within facility | | | | | 1 |  |
|  |  | Refers to another facility | | | | | 2 |  |
|  |  | Newborn died | | | | | 3 |  |
| 536 | Explains to mother (and support person) what happened | | 1 | | 2 | | 8 |  |
| 537 | Listens to mother and responds attentively to her questions and concerns | | 1 | | 2 | | 8 |  |
| 538 | *Observer: Did you call for help or intervene during the resuscitation to save the life of newborn?* | | 1 | | 2 | |  |  |
| **End of section 5; Please go to section 6** | | | | | | | | |

**Section 6: Payment for services**

| **No** | **Question & filters** | | **Yes** | **No** | **DK** | **Skip** |
| --- | --- | --- | --- | --- | --- | --- |
| 601 | Are women supposed to pay for maternity services? | | 1 | 2 | 8 |  |
| 602 | Are families supposed to pay for sick newborn care? | | 1 | 2 | 8 |  |
| 603 | Did the woman or her family pay any money for the services provided in the facility? | | 1 | 2 | 8 | No/DK🡪605 |
| 604 | How much did the woman pay in total for the since entering the facility till discharge? (In Taka) |  | | | |  |
| 605 | Did woman or family complain for money being charged? | | 1 | 2 | 8 |  |
| 606 | Was any service or supply withheld due to inability to pay? | | 1 | 2 | 8 |  |
| **End of section 6; Please go to section 7** | | | | | | |

**Section 7: Infection prevention and control**

| **No** | **Question & filters** | | **Yes** | **No** | | **DK** | **Skip** |
| --- | --- | --- | --- | --- | --- | --- | --- |
| 701 | Observe whether skilled attendants wear personal protective equipment (PPE) (Mask, cap, apron, gloves) | | 1 | 2 | | 8 |  |
| 702 | Is staff observed to be using PPE correctly? | | 1 | 2 | | 8 |  |
| 703 | Puncture-proof sharp container located in all clinical areas? | | 1 | 2 | | 8 | No/DK🡪705 |
| 704 | Are sharp containers less than ¾ full? | | 1 | 2 | | 8 |  |
| 705 | Is there a concrete-lined pit/ incinerator for sharp disposal? | | 1 | 2 | | 8 |  |
| 706 | Is there a well-ventilated, maintained and protected placenta pit (fenced)? | | 1 | 2 | | 8 |  |
| 707 | Did staff wash hands before and after examining patients? | | 1 | 2 | | 8 | No/DK🡪 709 |
| 708 | How do staff dry their hands after washing? | With clean regular towels | | | | 1 |  |
|  |  | Disposable towels | | | | 2 |  |
|  |  | Air dry | | | | 3 |  |
|  |  | Didn’t dry hands | | | | 4 |  |
|  |  | Others (specify)________ | | | | 7 |  |
| 709 | Did staff wear gloves when handling medical waste? | | 1 | | 2 | 8 |  |
| 710 | Was the delivery unit cleaned after the delivery? | | 1 | | 2 | 8 |  |
| 711 | Are functioning sinks with clean, running water available in following clinical areas for hand-washing? (one per area) *record ‘8’ if any area is absent* | | | | | | |
|  | 1. Emergency room | | 1 | | 2 | 8 |  |
|  | 1. Labour room/ward/area | | 1 | | 2 | 8 |  |
|  | 1. Delivery room/ area | | 1 | | 2 | 8 |  |
|  | 1. Post-operative ward | | 1 | | 2 | 8 |  |
|  | 1. Female ward | | 1 | | 2 | 8 |  |
| 712 | Are sinks equipped with bar/liquid soap in following clinical areas? *record ‘8’ if any area is absent* | | | | | | |
|  | 1. Emergency room | | 1 | | 2 | 8 |  |
|  | 1. Labour room/ward/area | | 1 | | 2 | 8 |  |
|  | 1. Delivery room/ area | | 1 | | 2 | 8 |  |
|  | 1. Post-operative ward | | 1 | | 2 | 8 |  |
|  | 1. Female ward | | 1 | | 2 | 8 |  |
| 713 | Are HW compliant with hand hygiene “five moments” | | 1 | | 2 | 8 |  |
| **End of section 7; If no complication of mother GO to section 10 (PNC)** | | | | | | | |

**Five moment:** Before touching a patient, before clean/aseptic procedure, after body fluid exposure,

After touching a patient, after touching patient’s surroundings

**Section 8: Observation of Postpartum Haemorrhage**

| *Record whether the provider carried out the following steps and/or examinations:*  *(some of the following steps may be performed simultaneously or by more than one provider)* | | | | | | | |
| --- | --- | --- | --- | --- | --- | --- | --- |
| **No** | **Question & filters** | **Yes** | **No** | **If Yes, record TIME/AMOUNT/DOSE** | | | **Skip** |
| 801 | Was this section observed? | 1 | 2 | Record time of observation starts | **:**  h h m m | | No🡪 Next section |
| 802 | Consent received from client (or proxy) if incapacitated | 1 | 2 | Record time of complication starts | **:**  h h m m | | No🡪 Next section |
| **Immediate care** | | | | | | | |
| 803 | Monitors bleeding | 1 | 2 | Amount (ml) |  | |  |
| 804 | Performs uterine massage | 1 | 2 | Record time | **:**  h h m m | |  |
| 805 | Gives oxytocin | 1 | 2 | Record time | **:**  h h m m | | No🡪807 |
|  |  | 1 | 2 | Record dose (IU) |  | |  |
| 806 | How oxytocin (IV) is administered? | Through ringer’s lactate | | | | 1 |  |
|  |  | Through normal saline | | | | 2 |  |
|  |  | Other (specify)_______________________ | | | | 7 |  |
| 807 | Any other uterotonic given? | 1 | 2 | Record time | **:**  h h m m | | No🡪809 |
| 808 | Which other uterotonic was given: | Ergometrine | | | | 1 |  |
|  |  | Syntometrin | | | | 2 |  |
|  |  | Misoprostol | | | | 3 |  |
|  |  | Other (specify)______________________ | | | | 7 |  |
| 809 | Examines vagina/perineum for lacerations/cervical tear | 1 | 2 | Record time | **:**  h h m m | |  |
| 810 | Examines placenta for completeness | 1 | 2 | Record time | **:**  h h m m | |  |
| 811 | Starts IV fluid | 1 | 2 | Record time | **:**  h h m m | |  |
| **Follow up care** | | | | | | | |
| 812 | Performs uterine exploration | 1 | 2 | Record time | **:**  h h m m | |  |
| 813 | Performs uterine mechanical evacuation | 1 | 2 | Record time | **:**  h h m m | |  |
| 814 | Performs manual removal of the placenta | 1 | 2 | Record time | **:**  h h m m | |  |
| 815 | Performs bimanual compression of the uterus | 1 | 2 | Record time | **:**  h h m m | |  |
| 816 | Performs aortic compression | 1 | 2 | Record time | **:**  h h m m | |  |
| 817 | Uses balloon or condom tamponed | 1 | 2 | Record time | **:**  h h m m | |  |
| 818 | Uses uterine sutures/catgut | 1 | 2 | Record time | **:**  h h m m | |  |
| 819 | Performs cardiac resuscitation | 1 | 2 | Record time | **:**  h h m m | |  |
| 820 | Sends/ refers to surgery for hysterectomy | 1 | 2 | Record time | **:**  h h m m | | If No🡪End section |
| 821 | Performs blood clotting time | 1 | 2 | Record time | **:**  h h m m | |  |
| 822 | Checks haemoglobin/ haematocrit | 1 | 2 | Record time | **:**  h h m m | |  |
| 823 | Requests blood grouping and cross matching | 1 | 2 | Record time | **:**  h h m m | |  |
| 824 | Gives blood products | 1 | 2 | Record time | **:**  h h m m | |  |
|  |  |  |  | Number of unit |  | |  |
| 825 | Gives antibiotics | 1 | 2 | Record time | **:**  h h m m | | No🡪827 |
| 826 | Which antibiotic was administered?  *(multiple answer possible)* | Penicillin | | | | A |  |
|  |  | Ampicillin | | | | B |  |
|  |  | Gentamicin | | | | C |  |
|  |  | Metronidazole | | | | D |  |
|  |  | Cephalosporin | | | | E |  |
|  |  | Other (specify)______________________ | | | | X |  |
| 827 | Gives additional dose of oxytocin | 1 | 2 | Record time | **:**  h h m m | | No🡪829 |
|  |  |  |  | Record dose (IU) |  | |  |
| 828 | Route of administration | IV | | | | 1 |  |
|  |  | IM | | | | 2 |  |
| 829 | Gives additional dose of other uterotonic | 1 | 2 | Record time | **:**  h h m m | | No🡪831 |
| 830 | Which other uterotonic was given:  *(Multiple answer possible)* | Ergometrine | | | | A |  |
|  |  | Syntometrin | | | | B |  |
|  |  | Misoprostol | | | | C |  |
|  |  | Other (specify)______________________ | | | | Y |  |
| 831 | Is the woman’s condition stable? | 1 | 2 | Record end time of observation | **:**  h h m m | |  |
| *If woman’s condition is not stable, continue observing until stable or for at least 1 hour after initial event* | | | | | | | |
| **Case details** | | | | | | | |
| 832 | What is the woman’s diagnosis | Atonic uterus | | | | A |  |
|  |  | Laceration | | | | B |  |
|  |  | Incomplete expulsion of placenta | | | | C |  |
|  |  | Placenta attached | | | | D |  |
|  |  | Coagulopathy | | | | E |  |
|  |  | Others (specify) | | | | Y |  |
|  |  | Don’t know | | | | Z |  |
| 833 | At what stage of labour and delivery did the complication occur: | Admitted with the complication | | | | 1 |  |
|  |  | At delivery | | | | 2 |  |
|  |  | Postpartum (before discharge) | | | | 3 |  |
|  |  | After discharge | | | | 4 |  |
| **End of section 8; If no other complication of mother END observation and record END time** | | | | | | | |

**Section 9: Observation of Severe Pre- eclampsia and Eclampsia**

|  | **Question & filters** | | | | | | | | **Yes** | | | **No** | | | **Skip** |
| --- | --- | --- | --- | --- | --- | --- | --- | --- | --- | --- | --- | --- | --- | --- | --- |
| 900 | Was this section observed? | | | | | | | | 1 | | | 2 | | | No🡪 Next section |
| 901 | Confirm that consent was received from client (or her proxy) | | | | | | | | 1 | | | 2 | | |  |
| 902 | Record time when complication started | | | | | | | |  | | | | | | |
| *Record whether the provider carried out the following steps and/or examinations: (some of the following steps may be performed simultaneously or by more than one provider).* | | | | | | | | | | | | | | | |
| **Immediate care** | | | | | | | | | | | | | | | |
| 903 | Blood pressure taken | | | | | | | | 1 | | | 2 | | No🡪 907 | |
| 904 | Record blood pressure: systolic (mm Hg) | | | | | | | |  | | | | |  | |
| 905 | Record blood pressure: diastolic (mm Hg) | | | | | | | |  | | | | |  | |
| 906 | Record time when blood pressure taken | | | | | | | | **:**  h h m m | | | | |  | |
| 907 | Urine checked for protein | | | | | | | | 1 | | | 2 | | No🡪910 | |
| 908 | Record result *(write down “+”; “++”; “+++”)* | | | | | Mild/ + | | | | | | | 1 |  | |
|  |  |  |  |  |  | Moderate/ ++ | | | | | | | 2 |  |  |
|  |  |  |  |  |  | Severe/ +++ | | | | | | | 3 |  |  |
| 909 | Record time when urine checked | | | | | | | | **:**  h h m m | | | | |  | |
| 910 | Were IV fluids started? | | | | | | | | 1 | | | 2 | | No🡪 912 | |
| 911 | Record time when IV fluids given | | | | | | | | **:**  h h m m | | | | |  | |
| 912 | Magnesium sulphate given **intramuscularly (IM)** | | | | | | | | 1 | | | 2 | | No🡪 915 | |
| 913 | Record dose *(in grams)* | | | | | | | |  | | | | |  | |
| 914 | Record time when magnesium-sulphate given IM | | | | | | | | **:**  h h m m | | | | |  | |
| 915 | Magnesium sulphate given **intravenously (IV)** | | | | | | | | 1 | | | 2 | | No🡪 918 | |
| 916 | Record dose *(in mL)* | | | | | | | |  | | | | |  | |
| 917 | Record time when magnesium sulphate given IV | | | | | | | | **:**  h h m m | | | | |  | |
| 918 | Diazepam given | | | | | | | | 1 | | | 2 | | No🡪922 | |
| 919 | Record dose *(in mg)* | | | | | | | |  | | | | |  | |
| 920 | Diazepam given intravenously (IV)? | | | | | | | | 1 | | | 2 | |  | |
| 921 | Record time when diazepam given | | | | | | | |  | | | | |  | |
| 922 | Antihypertensive given | | | | | | | | 1 | | | 2 | | No🡪 927 | |
| 923 | Which antihypertensive was given: | | Hydralazine/ Apresoline | | | | | | | | | 1 | |  | |
|  |  |  | Nifedipine | | | | | | | | | 2 | |  | |
|  |  |  | Labetolol | | | | | | | | | 3 | |  | |
|  |  |  | Methyledopa/ Aldomet | | | | | | | | | 4 | |  | |
|  |  |  | Other (specify) ______________ | | | | | | | | | 7 | |  | |
| 924 | Record dose *(in mg)* | | | | | | | |  | | | | |  | |
| 925 | Route of administration | | | Intramuscular (IM) | | | | | | | | 1 | | 1 | |
|  |  |  |  | Intravenous (IV) | | | | | | | | 2 | | 2 | |
|  |  |  |  | Oral/ sublingual | | | | | | | | 3 | |  | |
|  |  |  |  | Other (specify) ______________ | | | | | | | | 7 | | 3 | |
| 926 | Record time when antihypertensive given | | | | | | | | **:**  h h m m | | | | |  | |
| 927 | Urinary catheter placed | | | | | | | | 1 | | | 2 | | No🡪929 | |
| 928 | Record time when catheter placed | | | | | | | | **:**  h h m m | | | | |  | |
| 929 | Labour induced/augmented (incl. artificial membrane rupture) | | | | | | | | 1 | | | 2 | | No🡪931 | |
| 930 | Record time when labour induced or augmented | | | | | | | | **:**  h h m m | | | | |  | |
| 931 | Sent/referred to surgery for C-section | | | | | | | | 1 | | | 2 | | No🡪933 | |
| 932 | Record time when sent/referred to surgery for C-section | | | | | | | | **:**  h h m m | | | | |  | |
| 933 | Gives additional dose of magnesium sulphate | | | | | | | | 1 | | | 2 | | No🡪937 | |
| 934 | Record the additional dose *(in grams)* | | | | | | | |  | | | | |  | |
| 935 | Magnesium sulphate given intramuscular (IM)? | | | | | | | | 1 | | | 2 | |  | |
| 936 | Record time when magnesium sulphate given | | | | | | | | **:**  h h m m | | | | |  | |
| 937 | Gives additional dose of other medication | | | | | | | | 1 | | | 2 | | No🡪942 | |
| 938 | Which medication(s) was the client given *(circle all that apply)* | Diazepam | | | | | | | | | | A | | 🡪939 | |
|  |  | Hydralazine/ Apresoline | | | | | | | | | | B | | 🡪942 | |
|  |  | Nifedipine | | | | | | | | | | C | |  |  |
|  |  | Labetalol | | | | | | | | | | D | |  |  |
|  |  | Methyldopa/ Aldomet | | | | | | | | | | E | |  |  |
|  |  | Other (specify) ______________ | | | | | | | | | | Z | |  |  |
| 939 | Record dose for diazepam *(in mg)* | | | | | | | |  | | | | |  | |
| 940 | Diazepam given intravenously (IV)? | | | | | | | | 1 | | | 2 | |  | |
| 941 | Time diazepam given | | | | | | | | **:**  h h m m | | | | |  | |
| 942 | Calcium gluconate given | | | | | | | | 1 | | | 2 | | No🡪 944 | |
| 943 | Time calcium gluconate given | | | | | | | | **:**  h h m m | | | | |  | |
| **Monitoring** | | | | | | | | | | | | | | | |
| 944 | How many times following was checked/measured within 1st hour | | | | **Blood pressure** | | | **Reflexes** | | | **Respiration** | | |  | |
|  | Once | | | | 1 | | | 1 | | | 1 | | |  | |
|  | Twice | | | | 2 | | | 2 | | | 2 | | |  | |
|  | Three times | | | | 3 | | | 3 | | | 3 | | |  | |
|  | Four times | | | | 4 | | | 4 | | | 4 | | |  | |
|  | Five or more | | | | 5 | | | 5 | | | 5 | | |  | |
|  | Not checked/ measured during first hour | | | | 9 | | | 9 | | | 9 | | |  | |
| 945 | Is the woman’s condition stable? | | | | | | | 1 | | | 2 | | |  | |
| *If woman’s condition is not stable, continue observing until stable or for at least 1 hour after initial event* | | | | | | | | | | | | | | | |
| 946 | End time of observation | | | | | | **:**  h h m m | | | | | | |  | |
| **Case details** | | | | | | | | | | | | | | | |
| 947 | What is the woman’s diagnosis | Eclampsia | | | | | | | | | | 1 | |  | |
|  |  | Severe pre- eclampsia | | | | | | | | | | 2 | |  | |
|  |  | Other (specify)____________________ | | | | | | | | | | 7 | |  | |
| 948 | Was the woman unconscious | | | | | | 1 | | | 2 | | | |  | |
| 949 | Did the woman experience convulsion | | | | | | 1 | | | 2 | | | |  | |
| 950 | At what stage of labour and delivery did the complication occur | Before labour (including referral from ANC) | | | | | | | | 1 | | | |  | |
|  |  | During labour | | | | | | | | 2 | | | |  | |
|  |  | At delivery | | | | | | | | 3 | | | |  | |
|  |  | Postpartum (before discharge) | | | | | | | | 4 | | | |  | |
|  |  | After discharge | | | | | | | | 5 | | | |  | |
| **End of section 9** | | | | | | | | | | | | | | | |

**Section 10: Observation of first PNC before discharge of the mother and baby**

| **No** | **Questions and filters** | | | | | **Option/code** | | | | | | | **Skip** |
| --- | --- | --- | --- | --- | --- | --- | --- | --- | --- | --- | --- | --- | --- |
|  |  |  |  |  |  | **Yes** | **No** | | | **DK** | | |  |
| P00 | Was this section observed? | | | | | 1 | 2 | | |  | | | Yes🡪P02 |
| P01 | *If No, mention the reason* |  | | | | | | | | | | | End observation |
| P02 | Record the time of first postnatal contact  with any health worker | | | | | **:**  h h m m | | | | | | |  |
| P03 | Who conducted the first post-natal assessment | | Doctor | | | | | | | | | 1 |  |
|  |  |  | Midwife | | | | | | | | | 2 |  |
|  |  |  | Nurse | | | | | | | | | 3 |  |
|  |  |  | Untrained nurse(no EOC/ no midwifery training) | | | | | | | | | 4 |  |
|  |  |  | Others (specify)_________________________ | | | | | | | | | 7 |  |
| *Record whether the Health worker (HW) carried out the following steps and / or examinations:*  *(some of the following steps may be performed simultaneously or by another provider)* | | | | | | | | | | | | | |
| P04 | HW greets woman (and her baby) in respectful manner | | | | | 1 | | 2 | | | 8 | |  |
| P05 | Asks if mother is having any problem | | | | | 1 | | 2 | | | 8 | |  |
| P06 | Conducts any of the following procedures/assessment for the mother | | | | | | | | | | | |  |
|  | 1. Checks Pulse | | | | | 1 | | 2 | | | 8 | |  |
|  | 1. Measures Blood pressure | | | | | 1 | | 2 | | | 8 | |  |
|  | 1. Measures Temperature by thermometer | | | | | 1 | | 2 | | | 8 | |  |
|  | 1. Abdominal examination by palpation | | | | | 1 | | 2 | | | 8 | |  |
|  | 1. Checks pad for excessive bleeding / discharge | | | | | 1 | | 2 | | | 8 | |  |
|  | 1. Checks for anaemia | | | | | 1 | | 2 | | | 8 | |  |
|  | 1. Checked perineum / episiotomy for perineal trauma | | | | | 1 | | 2 | | | 8 | |  |
|  | 1. Assesses urine output | | | | | 1 | | 2 | | | 8 | |  |
|  | 1. Assesses bowel movement | | | | | 1 | | 2 | | | 8 | |  |
| P07 | Asks if mother has started breastfeeding (BF) yet | | | | | 1 | | 2 | | | 8 | | No/DK🡪P10 |
| P08 | Asks if mother is having any problem regarding BF baby | | | | | 1 | | 2 | | | 8 | |  |
| P09 | Assesses breastfeeding technique (position and attachment) | | | | | 1 | | 2 | | | 8 | |  |
| P10 | Shows mother appropriate position and attachment for BF | | | | | 1 | | 2 | | | 8 | |  |
| P11 | Did the HW diagnose a complication? | | | | | 1 | | 2 | | | 8 | | No/DK🡪P13 |
| P12 | Write the diagnosis? | | __________________________________________ | | | | | | | | | |  |
| P13 | Did the HW give the mother any medication? | | | | | 1 | | 2 | | | 8 | | No/DK🡪P17 |
| P14 | Record the medications and doses given to the mother: | | | | | | | | | | | | |
| a | Medication # 1 | | | a1: Name _________________ | | a2: Dose:_____________ | | | | | | | |
| b | Medication # 2 | | | b1: Name _________________ | | b2: Dose:____________ | | | | | | | |
| P15 | Did the HW explain how to use the medication? | | | | | 1 | | 2 | | | 8 | |  |
| P16 | Did the HW explain side effects of the medication? | | | | | 1 | | 2 | | | 8 | |  |
| P17 | HW asks mother if baby is having any problem | | | | | 1 | | 2 | | | 8 | |  |
| P18 | HW asks/discusses baby’s birth weight with the mother | | | | | 1 | | 2 | | | 8 | |  |
| P19 | Conducts any of the following procedures/assessment for the newborn | | | | | | | | | | | |  |
|  | 1. Assesses if baby had any convulsion | | | | | 1 | | 2 | | | 8 | |  |
|  | 1. Assesses the baby’s skin thoroughly | | | | | 1 | | 2 | | | 8 | |  |
|  | 1. Takes baby’s temperature | | | | | 1 | | 2 | | | 8 | |  |
|  | 1. Checks and counts breathing | | | | | 1 | | 2 | | | 8 | |  |
|  | 1. Checks heart rate | | | | | 1 | | 2 | | | 8 | |  |
|  | 1. Observed (or asked about) baby breastfeeding | | | | | 1 | | 2 | | | 8 | |  |
|  | 1. Inspected the cord stump | | | | | 1 | | 2 | | | 8 | |  |
| P20 | HW counsels mother on following for mother and baby | | | | | | | | | | | |  |
|  | 1. Mother should stay at facility for at least 24 hours | | | | | 1 | | 2 | | | 8 | |  |
|  | 1. Exclusive breastfeeding for 6 months | | | | | 1 | | 2 | | | 8 | |  |
|  | 1. Baby should be kept warm | | | | | 1 | | 2 | | | 8 | |  |
|  | 1. Baby should be kept with mother in the same room | | | | | 1 | | 2 | | | 8 | |  |
|  | 1. Bathing of baby should be delayed up to 72 hours | | | | | 1 | | 2 | | | 8 | |  |
|  | 1. Apply chlorhexidine to cord stump | | | | | 1 | | 2 | | | 8 | |  |
|  | 1. Importance of completing the vaccination for baby | | | | | 1 | | 2 | | | 8 | |  |
|  | 1. Advise mother on when to come for postnatal consultation | | | | | 1 | | 2 | | | 8 | |  |
|  | 1. Seek care if any danger sign for mother occurs | | | | | 1 | | 2 | | | 8 | |  |
|  | 1. Seek care if any danger sign for newborn occurs | | | | | 1 | | 2 | | | 8 | |  |
|  | 1. Advices on inter-pregnancy intervals (2 year interim) | | | | | 1 | | 2 | | | 8 | |  |
| P21 | HW counsels mother on following danger signs of the baby | | | | | | | | | | | |  |
|  | 1. Poor feeding or sucking | | | | | 1 | | 2 | | | 8 | |  |
|  | 1. Sleeping all the time | | | | | 1 | | 2 | | | 8 | |  |
|  | 1. Fever or hypothermia | | | | | 1 | | 2 | | | 8 | |  |
|  | 1. No stool by third day | | | | | 1 | | 2 | | | 8 | |  |
|  | 1. Blueness of the lips or skin | | | | | 1 | | 2 | | | 8 | |  |
|  | 1. Persistent vomiting/ vomiting with a swollen abdomen | | | | | 1 | | 2 | | | 8 | |  |
|  | 1. Difficulty establishing regular breathing | | | | | 1 | | 2 | | | 8 | |  |
|  | 1. Eye discharge | | | | | 1 | | 2 | | | 8 | |  |
|  | 1. Watery or dark green stools with mucus or with blood | | | | | 1 | | 2 | | | 8 | |  |
| P22 | HW counselled on postpartum family planning by asking | | | | | | | | | | | |  |
|  | 1. If she wants more children in the future? | | | | | 1 | | 2 | | | 8 | |  |
|  | 1. If she plans on exclusively breastfeeding her infant? | | | | | 1 | | 2 | | | 8 | |  |
|  | 1. If she has already chosen a family planning (FP) method | | | | | 1 | | 2 | | | 8 | |  |
| P23 | HW discusses FP methods with mother (or husband) | | | | | | | | | | | |  |
|  | 1. PPIUCD and interval IUCD | | | | | 1 | | 2 | | | 8 | |  |
|  | 1. Implant | | | | | 1 | | 2 | | | 8 | |  |
|  | 1. Injectable | | | | | 1 | | 2 | | | 8 | |  |
|  | 1. Oral contraceptives/ pill | | | | | 1 | | 2 | | | 8 | |  |
|  | 1. Condom | | | | | 1 | | 2 | | | 8 | |  |
|  | 1. Vasectomy | | | | | 1 | | 2 | | | 8 | |  |
|  | 1. Bilateral tubal ligation (BLTL) | | | | | 1 | | 2 | | | 8 | |  |
| P24 | Did the HW provide any family planning method to mother? | | | | | 1 | | 2 | | | 8 | |  |
| P25 | Did the HW provide any immunizations to the baby? | | | | | 1 | | 2 | | | 8 | |  |
| P26 | HW used visual aids during consultation | | | | | 1 | | 2 | | | 8 | |  |
| P27 | Record information on a patient card or register | | | | | 1 | | 2 | | | 8 | |  |
| P28 | Wash hands with soap/hand sanitizer before examining baby | | | | | 1 | | 2 | | | 8 | |  |
| P29 | Wash hands with soap/hand sanitizer after examining baby | | | | | 1 | | 2 | | | 8 | |  |
| **Outcome before discharge** | | | | | | | | | | | | | |
| P30 | Record outcome of visit | | | | Mother | | | | Baby | | | |  |
|  | Goes home | | | | 1 | | | | 1 | | | |  |
|  | Referred to other department of same facility | | | | 2 | | | | 2 | | | |  |
|  | Referred to other facility | | | | 3 | | | | 3 | | | |  |
|  | Started first dose of antibiotic | | | |  | | | | 4 | | | |  |
|  | Don’t know | | | | 8 | | | | 8 | | | |  |
| **END observation and record END time** | | | | | | | | | | | | | |

**Structured observation of PNC**

**(Supplement form for delivery observation Section 10)**

| **No** | **Questions and filters** | **Yes** | **No** | **DK** | **Skip** |
| --- | --- | --- | --- | --- | --- |
| S00 | Was this section observed? | 1 | 2 |  | No🡪 skip the section |
| S01 | HW introduced her / himself and title (doctor, nurse) | 1 | 2 | 8 |  |
| S02 | HW explains to mother about what is exclusive breastfeeding | 1 | 2 | 8 |  |
| S03 | HW talked about importance of exclusive breastfeeding | 1 | 2 | 8 |  |
| S04 | HW mentions how long baby should be exclusively breastfed | 1 | 2 |  | No🡪S06 |
| S05 | Record, how long was mentioned (in months) |  | | |  |
| S06 | HW mentions about appropriate frequency of breastfeeding | 1 | 2 |  | No🡪S08 |
| S07 | Record, frequency mentioned (number of times/day) |  | | |  |
| S08 | HW mentions about duration of each session of breastfeeding | 1 | 2 |  | No🡪S10 |
| S09 | Record, duration mentioned (in minutes) |  | | |  |
| S10 | Mentioned overall duration of breast feeding? | 1 | 2 | 8 | No🡪S12 |
| S11 | Record, overall duration mentioned (in hours) |  | | |  |
| S12 | Circle appropriate code for following if mentioned by HW regarding POSITIONING of breast? | | | | |
|  | a. Baby’s ear shoulder and hip is placed straight | 1 | 2 | 8 |  |
|  | b. Baby’s face is facing breast with nose opposite to nipple | 1 | 2 | 8 |  |
|  | c. Baby’s body is held close to the mother | 1 | 2 | 8 |  |
|  | d. Baby’s whole body is supported | 1 | 2 | 8 |  |
| S13 | Circle appropriate code for following if mentioned by HW regarding ATTACHMENT of breast? | | | | |
|  | a. Chin is touching the breast | 1 | 2 | 8 |  |
|  | b. Mouth is wide open | 1 | 2 | 8 |  |
|  | c. Lower lip is turned outwards | 1 | 2 | 8 |  |
|  | d. More areola is visible above than below the mouth | 1 | 2 | 8 |  |
| S14 | Circle appropriate code for following if mentioned by HW regarding effective suckling | | | | |
|  | a. Slow deep sucks | 1 | 2 | 8 |  |
|  | b. Occasional short pauses | 1 | 2 | 8 |  |
| S15 | HW discouraged mother regarding bottle feeding | 1 | 2 | 8 |  |
| S16 | HW discouraged mother regarding any teats/ pacifiers | 1 | 2 | 8 |  |
| S17 | HW discouraged commercially available formula feeding | 1 | 2 | 8 |  |
| S18 | Circle appropriate code for following if mentioned by HW regarding signs that baby is not getting enough milk | | | | |
|  | a. Poor weight gain | 1 | 2 | 8 |  |
|  | b. Passing small amount of concentrated urine(<6 times/day) | 1 | 2 | 8 |  |
|  | c. Baby not satisfied after breastfeeding/ Prolonged BF | 1 | 2 | 8 |  |
|  | d. Baby cries often | 1 | 2 | 8 |  |
|  | e. Very frequent breastfeeding | 1 | 2 | 8 |  |
|  | f. Baby refuses to breastfeeding | 1 | 2 | 8 |  |
|  | g. Baby passes hard, dry or green stools | 1 | 2 | 8 |  |
|  | h. No milk comes when mother tries to express | 1 | 2 | 8 |  |
| S19 | Circle appropriate code for following if advised by HW on what mother should do when there is not enough milk? | | | | |
|  | a. To correct positioning and attachment during BF | 1 | 2 | 8 |  |
|  | b. To check baby for illness, abnormality, growth and take to health facility | 1 | 2 | 8 |  |
|  | c. To examine the mother and her breasts for any problems | 1 | 2 | 8 |  |
|  | d. To eat more food | 1 | 2 | 8 |  |
|  | e. To keep hydrated | 1 | 2 | 8 |  |
|  | f. To build confidence and seek support | 1 | 2 | 8 |  |
| S20 | HW talked about proper formulation of cow’s milk or proper way to give formula in case breast-milk is absolutely unavailable | 1 | 2 | 8 |  |
| S21 | HW told mother about appropriate age of initiation of complementary feeding? | 1 | 2 |  | No🡪 S23 |
| S22 | If yes, mention the age (in months) |  | | |  |
| S23 | HW asked mother if she had any more questions to clarify regarding breastfeeding the baby | 1 | 2 | 8 |  |
| **END observation and record END time** | | | | | |
