# Supplementary material for: Initiation of breastfeeding within one hour of birth and its determinants among normal vaginal deliveries at primary and secondary health facilities in Bangladesh: A case-observation study
Source: PLoS One. 2018 Aug 16;13(8):e0202508. doi: 10.1371/journal.pone.0202508 (PMC6095597; doi:10.1371/journal.pone.0202508)
Supplement: S2 Appendix — (DOCX) [file pone.0202508.s002.docx]

**S2 Appendix: Exit interview of women and family members**

**Objective of the interview:**

To assess their satisfaction about the services they received at the facility. The major domains of client satisfaction will include right based care; communication; respect, privacy and dignity; emotional and psychological support.

**Instruction for the data collectors**

- The data will be collected principally by interviewing the women who just had their delivery done in that specific facility and family members of recently delivered mother.
- Data collector will take written consent must before starting the interview and will be concerned about interviewees privacy and willingness.
- The data collectors should pay specific attention to the **SKIP** questions
- Please check for completeness of the form before ending of the interview

**_______________________________________________________________________________**

**Date of interview**: **-****-** **Time of onset:** : **(24 hour)**

**D D |M M | Y Y Y Y**

**Name of the Interviewer’s: ______________________________ Code:**

1. **Information about facility identification:**

|  | **Name** | **Code** |
| --- | --- | --- |
| স্বাস্থ্যকেন্দ্র Facility | --------------------------------------- |  |
| স্বাস্থ্যকেন্দ্রের ঠিকানা **Address of the facility:** | | |
| জেলা District | --------------------------------------- |  |
| উপজেলা Upazila | --------------------------------------- |  |
| স্বাস্থ্যকেন্দ্রের ধরণ Type of the facility | জেলা হাসপাতাল/ মেডিকেল কলেজ হাসপাতাল District hospital/ Medical College and hospital | 1 |
|  | উপজেলা স্বাস্থ্যকেন্দ্র Upazila Health Complex | 2 |
| **মা-নবজাতক জরূরী ব্যবস্থাপনা** Facility Designation | BEmONC | 1 |
|  | CEmONC | 2 |
| **স্বাস্থ্যকেন্দ্রের** মালিকানা Facility Ownership | **সরকারী** Government | 1 |
|  | প্রাইভেট Private | 2 |
|  | বেসরকারী অলাভজনক প্রতিষ্ঠান Faith Based Organization (FBO) | 3 |

1. **Client information**

| **No** | **Points & questions** | **Options** | | **Coding** | **skip** |
| --- | --- | --- | --- | --- | --- |
| B1 | রোগীর নাম ও কোড Client ID | ___________________ | |  |  |
| B2 | বয়স Age (in years) |  | |  |  |
| B3 | এই গর্ভ সহ মোট গর্ভের সংখ্যা Number of all previous pregnancies (including this) |  | |  |  |
| B4 | এই ডেলিভারী সহ মোট ডেলিভারীর সংখ্যা Number of deliveries (including this delivery) |  | |  |  |
| B5 | আপনি কি পড়তে পারেন? Literate: Can you read: | হ্যাঁ Yes | | 1 |  |
|  |  | না No | | 2 |  |
| B6 | আপনি কি লিখতে পারেন? Literate: Can you write: | হ্যাঁ Yes | | 1 |  |
|  |  | না No | | 2 |  |
| B7 | আপনি কি কখনো স্কুলে/ মাদ্রাসায়/ উপানুষ্ঠানিক শিক্ষা স্কুলে (বয়স্ক শিক্ষা কেন্দ্র/ অন্য কোথাও) লেখাপড়া করেছেন? Did you ever study in school/ madrasa/ non formal school? | হ্যাঁ Yes | | 1 |  |
|  |  | না No | | 2 | 🡪B9 |
| B8 | শিক্ষাগত যোগ্যতা Education level | **প্রাথমিক** অসম্পূর্ন Primary incomplete | | 1 |  |
|  |  | **প্রাথমিক** সম্পূর্ন primary complete | | 2 |  |
|  |  | মাধ্যমিক Secondary / Vocational/ Technical | | 6 |  |
|  |  | উচ্চ-মাধ্যমিক Post Secondary Education | | 3 |  |
|  |  | ব্যাচেলর এবং অনার্স/ Bachelor with honors | | 4 |  |
|  |  | মাস্টার্স অথবা উচতর ডিগ্রী/ Masters or Higher | | 5 |  |
| B9 | আপনার ধর্ম Your religion | ইসলাম Islam | | 1 |  |
|  |  | হিন্দু Hindu | | 2 |  |
|  |  | বৌদ্ধ Buddhism | | 3 |  |
|  |  | খ্রিষ্টান Christianity | | 4 |  |
|  |  | অন্যান্য (নির্দিষ্ট করুন) others (specify)  _____________________________ | | 7 |  |
| B10 | আপনার পেশা কি? What is your occupation? | অদক্ষ কর্মী (কামলা, মাটি কাটা) Unskilled labourer | | 1 |  |
|  |  | দক্ষ কর্মী (কাঁথা সেলাই, দরজির কাজ, বুটিকের কাজ ইত্যাদি) Skilled worker | | 2 |  |
|  |  | ব্যবসা Business | | 3 |  |
|  |  | চাকুরীজীবি Service holder | | 4 |  |
|  |  | পেশাজীবি (ইঞ্জিনিয়ার/ডাক্তার/ শিক্ষক/ উকিল) Professional | | 5 |  |
|  |  | Housewife গৃহিণী | | 6 |  |
|  |  | অন্যান্য (নির্দিষ্ট করুন) Other (specify)  _____________________________ | | 7 |  |
| B11 | আপনার গর্ভের সময়ই কি আপনি ঠিক করেছিলেন যে এই স্বাস্থ্যকেন্দ্রে প্রসব করাবেন? Did you plan during the pregnancy to deliver in this facility? | হ্যাঁ, সব প্রসব পূর্ববর্তী সেবায় অংশ নিয়েছি Yes, always attended ANC here | | 1 |  |
|  |  | হ্যাঁ, আমি এই স্বাস্থ্যকেন্দ্রে আসা পছন্দ করি Yes, because I like here | | 2 |  |
|  |  | না, অন্য স্বাস্থ্যকেন্দ্র থেকে জরুরী ভিত্তিতে পাঠিয়েছে No, was referred from another facility for emergency | | 3 |  |
|  |  | না, বাড়ি থেকে জটিলতার কারণে জরুরী ভিত্তিতে এখানে এনেছে No, was brought in an emergency from home | | 4 |  |
| B12 | প্রসবের ধরণ Kind of delivery | স্বাভাবিক প্রসব Spontaneous vaginal delivery | | 1 | 🡪 B16 |
|  |  | এসিস্টেড প্রসব(ভ্যাক্যুম অথবা ফরসেপ) Assisted vaginal delivery (vacuum or forceps) | | 2 | 🡪 B16 |
|  |  | পরিকল্পিত সিজারিয়ান সেকশন A planned caesarean delivery | | 3 | 🡪 B14 |
|  |  | জরুরী সিজারিয়ান সেকশন An emergency caesarean delivery | | 4 |  |
| B13 | সিজারিয়ান সেকশন করার আগে তারা কি আপনাকে মত দিতে জিজ্ঞাসা করেছিল? Did they ask for you to agree before the operation was done? | হ্যাঁ, আমি মত দিয়েছি Yes, I agreed | | 1 |  |
|  |  | না, কিন্তু আমার সঙ্গী আমার পক্ষ হয়ে মত দিয়েছে No, but my caretaker agreed on my behalf | | 2 | 🡪 B15 |
|  |  | না, তারা কিছু না বলেই নিয়ে গেছে No, they just took me in; | | 3 | 🡪 B15 |
| B14 | সিজারিয়ান সেকশন করার আগে আপনার লিখিত সম্মতি নেওয়া হয়েছিলো? If you had a caesarean, was consent obtained from you before it was done? | হ্যাঁ Yes | | 1 |  |
|  |  | না No | | 2 |  |
| B15 | সিজারিয়ান সেকশন কেন করা হয়েছিল তা কি আপনাকে বলা হয়েছিল Have you been told why the caesarean was done? | হ্যাঁ Yes | | 1 |  |
|  |  | না No | | 2 |  |
| B16 | প্রসব এর সময় আপনার সাথে কাউকে থাকতে দেওয়া হয়েছিল? (যেমনঃ আপনার স্বামী, পরিবারের অন্যান্য সদস্য, বন্ধু) Were you allowed to have companion during labour? (*husband or family member or friend)* | হ্যাঁ Yes | | 1 |  |
|  |  | না No | | 2 | 🡪 B19 |
|  |  | জানা নেই/ মনে নেই Don’t know/ Can’t remember | | 8 | 🡪 B19 |
| B17 | আপনি কি তাকে নিজে বাছাই করেছিলেন? did you choose your companion | হ্যাঁ Yes | | 1 |  |
|  |  | না No | | 2 |  |
| B18 | কে সাথে ছিল? Who accompanied you? | স্বামী Husband | | 1 |  |
|  |  | মা/ শ্বাশুড়ি Mother/ mother in law | | 2 |  |
|  |  | আত্মীয় Relative | | 3 |  |
|  |  | বন্ধু/প্রতিবেশী Friend/neighbor | | 4 |  |
|  |  | অন্যান্য (নির্দিষ্ট করুন) Other (specify)  _____________________________ | | 7 |  |
| B19 | প্রসব-পরবর্তী সময় আপনার সাথে কাউকে থাকতে দেওয়া হয়েছিল? May you allowed to have companion during the postnatal period? | হ্যাঁ Yes | | 1 |  |
|  |  | না No | | 2 |  |
|  |  | জানা নেই/ মনে নেই Don’t know/ Can’t remember | | 8 |  |
| B20 | বাচ্চার ব্যাপারে কাউন্সেলিং করার সময় আপনার সঙ্গীকে ডেকেছিল? Was a companion called to be with you during counselling on the care of the baby? | হ্যাঁ Yes | | 1 |  |
|  |  | না No | | 2 |  |
|  |  | প্রযোজ্য নয় N/A | | 3 | 🡪B23 |
| B21 | প্রসবের কতক্ষণ পরে আপনি বাচ্চাকে বুকের দুধ খাওয়ানো শুরু করেছিলেন? After how long did you start to breastfeeding your baby? | ৩০ মিনিটের কম সময়ে less than 30mins | | 1 |  |
|  |  | ৩০- ৬০ মিনিটের মধ্যে 30-60 mins | | 2 |  |
|  |  | ১ ঘন্টা পর After 1 hour; | | 3 |  |
|  |  | বাচ্চাকে বুকে দেওয়া হয়নি not put to breast | | 4 |  |
| B22 | জন্মের ঠিক পরপরই কি বাচ্চাকে আপনার শরীরের সাথে লাগিয়ে রাখার জন্যে দিয়েছিল? Did the newborn put skin-to-skin with the mother immediately after birth? | হ্যাঁ Yes | | 1 |  |
|  |  | না No | | 2 |  |
|  |  | জানা নেই Don’t know | | 8 |  |
| B23 | আপনার প্রসবের পরে নিম্নোক্ত কোনো জটিলতা কি হয়েছিল? Did you have any of the following complications after the birth? | | **Yes** | **No** |  |
|  | 1. অতিরিক্ত রক্তক্ষরণ যাতে কাপড় ভিজে যায়, মেঝে ভেসে যায়, শরীরে রক্ত দিতে হয় অথবা পুনরায় অপারেশন করা লাগে *Excessive bleeding that wet your clothes, poured on the floor, needed transfusion with blood or needed operation again?* | | 1 | 2 |  |
|  | 1. খিঁচুনি হওয়া বা অজ্ঞান হয়ে যাওয়া *Collapsed or convulsed* | | 1 | 2 |  |
|  | 1. আপনার রক্তচাপ বেড়ে গিয়েছিল *You were told your BP was high* | | 1 | 2 |  |
|  | 1. আপনার অনেক জ্বর ছিল *You had a temperature or fever* | | 1 | 2 |  |

1. **Reception and triage**

| **No** | **Points & questions** | **Options** | | **Code** | **skip** |
| --- | --- | --- | --- | --- | --- |
| C1 | স্বাস্থ্যকেন্দ্রে আসার পর থেকে একজন সেবাদানকারীর দেখা পাওয়া পর্যন্ত আপনাকে কেমন সময় অপেক্ষা করতে হয়েছে? How long did you have to wait at the facility before you were first seen by a health professional when you first came in for this delivery? | ১০ মিনিটের কম less than 10mins | | 1 |  |
|  |  | ১০- ৩০ মিনিট 10-30mins | | 2 |  |
|  |  | ৩০- ৬০ মিনিট 30-60mins | | 3 |  |
|  |  | ১ ঘন্টার বেশি over 1 hour | | 4 |  |
| C2 | আপনাকে কি জিজ্ঞাসা করা হয়েছিল কেন আপনি স্বাস্থ্যকেন্দ্রে এসেছেন ও আপনার গর্ভ সম্পর্কে কোনো প্রশ্ন জিজ্ঞাসা করেছিল? Were you asked why you came and about your pregnancy at first arrival? | হ্যাঁ Yes | | 1 |  |
|  |  | না No | | 2 |  |
| C3 | স্বাস্থ্যকেন্দ্রে আসার আগেই আপনার পানি ভেঙ্গে গিয়েছিল কি? Did you break water before coming to facility? | হ্যাঁ Yes | | 1 |  |
|  |  | না No | | 2 | **🡪** C5 |
| C4 | স্বাস্থ্যকেন্দ্রে আসার কতক্ষণ আগে আপনার পানি ভেঙ্গে গিয়েছিল? For how long did your waters break before you came to the facility? | ১০ মিনিটের কম less than 10mins | | 1 |  |
|  |  | ১০- ৩০ মিনিট 10-30mins | | 2 |  |
|  |  | ৩০- ৬০ মিনিট 30-60mins | | 3 |  |
|  |  | ১ ঘন্টার বেশি over 1 hour | | 4 |  |
| C5 | স্বাস্থ্যকর্মী কি লেবার ওয়ার্ডে পাঠানোর আগে নিম্নোক্ত কাজগুলো করেছিল? (ঠিকমত তাকে ব্যাখ্যা করেন) Did the HW do any of the following before sending you to the labour ward (demonstrate to her as appropriate)? | | **Yes** | **No** |  |
|  | - 1. আপনার চোখের পাতা, জিহ্বা বা নখ পরীক্ষা করেছেন *Check your eyelids, tongue or nails* | | 1 | 2 |  |
|  | - 1. রক্তচাপ পরীক্ষা করেছিল *Checked your BP* | | 1 | 2 |  |
|  | - 1. প্রস্রাব নিয়েছিল ও পরীক্ষা করতে পাঠিয়েছিল *Took your urine and checked* | | 1 | 2 |  |
|  | - 1. পরীক্ষা করে দেখেছিল আপনার রক্তক্ষরণ হচ্ছে কিনা *Checked whether you were bleeding* | | 1 | 2 |  |
|  | - 1. আপনার পেট পরীক্ষা করেছিল *Examined your abdomen* | | 1 | 2 |  |
|  | - 1. বাচ্চার হৃদকম্পন শুনেছিল *Listen to baby's heart beats ?* | | 1 | 2 |  |
| C6 | আপনাকে কি তারা বলেছিল লেবার ওয়ার্ডে পাঠানোর আগে পরীক্ষা করে তারা কি পেয়েছিল? Did they tell what they found before sending you to labour ward? | হ্যাঁ Yes | | 1 |  |
|  |  | না No | | 2 |  |
| C7 | স্বাস্থ্যকেন্দ্রে প্রথম আসার পর স্বাস্থ্যকর্মীরা আপনার সাথে যেভাবে কথা বলেছে তাতে আপনার কি মনে হয়েছে? How would you describe the way the health workers talked to you in the facility when you first came in? | বন্ধুত্বপূর্ন Friendly | | 1 |  |
|  |  | সাধারণ Neutral | | 2 |  |
|  |  | মাঝে মাঝে বন্ধুত্বপূর্ন ছিলনা Often unfriendly | | 3 |  |
|  |  | কড়া ভাষা ছিল Harsh | | 4 |  |
| C8 | আপনি কি মনে করেন আপনার সাথে কথা বলার সময় তারা যথেষ্ট গোপনীয়তা মেনে চলেছে? Do you think they considered your privacy in their interactions with you? | হ্যাঁ Yes | | 1 |  |
|  |  | না No | | 2 |  |
| C9 | আপনি যখন কোনো প্রশ্ন করতে চেয়েছেন তারা তা করতে দিয়েছে? Did they allow you to ask questions when you wanted? | হ্যাঁ Yes | | 1 |  |
|  |  | না No | | 2 |  |
|  |  | কোনো প্রশ্ন করতে চাইনি did not want to ask any question | | 3 |  |
| C10 | আপনি এই স্বাস্থ্যকন্দ্রে থাকা অবস্থায় যেমন গোপনীয়তা পেয়েছেন তাতে কি আপনি সন্তুষ্ট? Are you satisfied with the level of privacy provided during your stay in the health facility? | হ্যাঁ Yes | | 1 |  |
|  |  | না No | | 2 |  |

1. **Labour ward and delivery :**

| **No** | **Points & questions** | **Options** | | | | **Coding** | **skip** |
| --- | --- | --- | --- | --- | --- | --- | --- |
| D1 | লেবার ওয়ার্ডে আসার পর আপনাকে স্বাস্থ্যকর্মী প্রথম দেখা পর্যন্ত আপনাকে কতক্ষণ অপেক্ষা করতে হয়েছে? How long did you have to wait at the labour ward before you were first seen by a health professional when you first came in for this delivery? | ১০ মিনিটের কম less than 10mins | | | | 1 |  |
|  |  | ১০- ৩০ মিনিট 10-30mins | | | | 2 |  |
|  |  | ৩০- ৬০ মিনিট 30-60mins | | | | 3 |  |
|  |  | ১ ঘন্টার বেশি over 1 hour | | | | 4 |  |
| D2 | লেবার ওয়ার্ডে থাকা অবস্থায় কতক্ষণ পর পর স্বাস্থ্যকর্মীরা আপনাকে দেখতে এসেছিল? How often did they come to check your labour whilst you were there? | আধা ঘন্টা পর পর half hourly | | | | 1 |  |
|  |  | এক ঘন্টা পর পর 1 hourly | | | | 2 |  |
|  |  | ২-৪ ঘন্টা পর পর 2-4hourly | | | | 3 |  |
|  |  | ৪-৬ ঘন্টা পর পর 4-6hourly | | | | 4 |  |
|  |  | ৬ ঘন্টার বেশি longer than 6 hourly | | | | 5 |  |
|  |  | প্রসবের আগ পর্যন্ত কোনো পরীক্ষা হয়নি Not examined again till delivery | | | | 6 |  |
| D3 | লেবারে থাকার সময় নিম্নোক্ত কোনো কিছু কি আপনাকে দেওয়া হয়েছিল? Were you given any of the following during your labour? | | হ্যাঁ Yes | না No | মনে নেই Can’t remember | |  |
|  | 1. আই ভি *IV fluid* | | 1 | 2 | 3 | |  |
|  | 1. ওষুধ *Medicine to take* | | 1 | 2 | 3 | |  |
|  | 1. ইনজেকশন *Injection (IM)* | | 1 | 2 | 3 | |  |
|  | 1. ব্যাথার ওষুধ *Pain relief* | | 1 | 2 | 3 | |  |
| D4 | সন্তান প্রসবের আগে কতক্ষণ আপনি তীব্র ব্যাথা ও জরায়ু সঙ্কোচন সহ্য করেছেন? For how long were you experiencing severe pains and contractions (been in active labour) before you delivered? | ৬ ঘন্টার কম সময় Less than 6 hrs | | | | 1 |  |
|  |  | ৬-৮ ঘন্টা 6-8 hours | | | | 2 |  |
|  |  | ৮- ১০ ঘন্টা 8-10 hours | | | | 3 |  |
|  |  | ১০- ১২ ঘন্টা 10- 12 hours | | | | 4 |  |
|  |  | ১২- ২৪ ঘন্টা 12-24hrs | | | | 5 |  |
|  |  | প্রযোজ্য নয় N/A | | | | 9 |  |
| D5 | কখনো কি আপনার মনে হয়েছে স্বাস্থ্যকর্মীরা আপনার ও আপনার বাচ্চার জন্যে যথেষ্ট পরিমান করছেনা? Was there any time that you were concerned that they were not doing enough for you and the baby? | হ্যাঁ Yes | | | | 1 |  |
|  |  | না No | | | | 2 |  |
| D6 | লেবার এবং প্রসবের সময় স্বাস্থ্যসেবাদানকারী আপনার সাথে কেমন সময় ব্যয় করেছিল? Was the time the health worker(s) spend with you during labour and delivery; | অনেক কম সময় too short | | | | 1 |  |
|  |  | মোটামুটি সময় just okay | | | | 2 |  |
|  |  | অনেক বেশি সময় too long | | | | 3 |  |
| D7 | প্রসবের পর থেকে আপনি কতক্ষণ স্বাস্থ্যকেন্দ্রে ছিলেন? (ঘন্টা, এবং প্রযোজ্য হলে দিনে রেকর্ড করুন) How long did you spend in the health facility after you delivered? (*record in hours and if applicable days)* | ঘন্টা hours  দিন days) | | | | 1  2 |  |
| D8 | আপনি লেবার রুম এবং প্রসবকালীন সময়ে যেমন গোপনীয়তা পেয়েছেন তাতে কি আপনি সন্তুষ্ট? Are you satisfied with the level of privacy provided during labour and delivery? | হ্যাঁ Yes | | | | 1 |  |
|  |  | না No | | | | 2 |  |
| D9 | ডেলিভারীর পর থেকে আপনার থাকাকালীন সময়টি কি আপনার সুবিধা অনুযায়ী ছিল? Were you happy with the time you stayed in the facility after delivery? | হ্যাঁ Yes | | | | 1 | 🡪E1 |
|  |  | না No | | | | 2 |  |
| D10 | যদি না হয়, বলুন আর কি সুবিধা আপনি চেয়েছিলেন? If No, what would you preferred? | আরো বেশি সময় থাকতে Stay longer | | | | 1 |  |
|  |  | আরো কম সময় থাকতে Stay shorter | | | | 2 |  |
|  |  | আরো বেশী তথ্য জানতে চেয়েছিলাম Receive more information | | | | 3 |  |
|  |  | অন্যান্য (নির্দিষ্ট করুন) Other (specify)  _________________________ | | | | 7 |  |

1. **Respectful and dignified care:**

| **No** | **Points & questions** | | **Yes** | | | | **No** | | **skip** |
| --- | --- | --- | --- | --- | --- | --- | --- | --- | --- |
| E1 | আপনার কি মনে হয় আপনি যখন কারো সাহায্য চেয়েছেন, ঠিকমত সাড়া পেয়েছেন? Do you think the health workers were responsive, when you ask for their support? | | 1 | | | | 2 | |  |
| E2 | আপনার কি মনে হয় স্বাস্থ্যকর্মীরা আপনাকে সম্মানের সাথে চিকিৎসা করেছেন? Do you think the health worker(s) treated you with respect? | | 1 | | | | 2 | |  |
| E3 | স্বাস্থ্যকেন্দ্রে থাকাকালীন সময়ে নিম্নোক্ত কোনো কিছুর সম্মুখীন কি আপনি হয়েছিলেন? Were you subjected to any of the following during your stay at the facility? | | | | | | | |  |
|  | 1. শারীরিক নির্যাতন Physical abuse | | 1 | | | | 2 | |  |
|  | 1. মৌখিক নির্যাতন Verbal abuse | | 1 | | | | 2 | |  |
|  | 1. যৌন নির্যাতন Sexual abuse | | 1 | | | | 2 | |  |
|  | 1. অন্যান্য (নির্দিষ্ট করুন) Other (specify)   _____________________________ | | 1 | | | | 2 | |  |
| E4 | নিম্নের বিষয়গুলো নিয়ে যদি আপনি কিছু শুনে থাকেন তা নিয়ে আপনি কি সন্তুষ্ট? Are you satisfied with the information you received about the following: | | হ্যাঁ **Yes** | না **No** | | | কিছু বলা হয়নি Not received | |  |
|  | a) বুকের দুধ খাওয়ানো Breastfeeding | | 1 | 2 | | | 3 | |  |
|  | b) প্রসব-পরবর্তী সেবা এবং পরিচ্ছন্নতা Postpartum care and hygiene | | 1 | 2 | | | 3 | |  |
|  | c) পরিবার পরিকল্পনা Family planning | | 1 | 2 | | | 3 | |  |
| E5 | প্রসবের পর মায়ের যেসকল বিপদ চিহ্ন দেখা দিলে দ্রুত ডাক্তার দেখানো উচিৎ তা কি আপনাকে বলা হয়েছে?? Were you told danger signs after childbirth for the mother - when you have to seek care in a health facility | হ্যাঁ Yes | | | | | | 1 |  |
|  |  | না No | | | | | | 2 | 🡪 E7 |
| E6 | আপনি কি সেই বিপদচিহ্নগুলোর নাম বলতে পারবেন? (মায়ের বলা প্রতিটি উত্তর বৃত্তায়িত করুন) Can you give/mention some of these danger signs?  *(****do not*** *read the options, tick the signs the woman mentions)* | | | | | Code | | |  |
|  | 1. মাসিকের রাস্তা দিয়ে রক্তক্ষরণ vaginal bleeding | | | | | A | | |  |
|  | 1. খিচুনি convulsions | | | | | B | | |  |
|  | 1. প্রচন্ড মাথা ব্যথা Severe headache | | | | | C | | |  |
|  | 1. প্রচন্ড দুর্বলতা Too weak to get out of bed | | | | | D | | |  |
|  | 1. প্রচন্ড পেটে ব্যথা severe abdominal pain | | | | | E | | |  |
|  | 1. জ্বর fever | | | | | F | | |  |
|  | 1. স্তন ফুলে যাওয়া, ব্যথা অথবা লাল হয়ে যাওয়া breast swollen, red or tender breasts, or sore nipple | | | | | G | | |  |
|  | 1. প্রস্রাব চুইয়ে পরা urine dribbling | | | | | H | | |  |
|  | 1. প্রস্রাবের সময়ে জ্বালাপোড়া Pain on Micturition | | | | | I | | |  |
|  | 1. দৃষ্টি ঝাপসা হয়ে আসা blurred vision | | | | | J | | |  |
|  | k) অন্যান্য (নির্দিষ্ট করুন) Other (specify)  _____________________________ | | | | | Y | | |  |
| E7 | প্রসবের পর **বাচ্চার যেসকল বিপদ চিহ্ন** দেখা দিলে দ্রুত ডাক্তার দেখানো উচিৎ তা কি আপনাকে বলা হয়েছে? Danger sign for newborn when you have to seek care in health facility | হ্যাঁ Yes | | | | | | 1 |  |
|  |  | না No | | | | | | 2 | 🡪 E9 |
| E8 | আপনি কি সেই বিপদচিহ্নগুলোর নাম বলতে পারবেন? (মায়ের বলা প্রতিটি উত্তর বৃত্তায়িত করুন) Can you give/mention some of these danger signs? *(****do not*** *read the options)* | | | | | | | Code |  |
|  | 1. শ্বাস নিতে কষ্ট difficulty in breathing | | | | | | | A |  |
|  | 1. খিচুনি convulsions | | | | | | | B |  |
|  | 1. জ্বর fever | | | | | | | C |  |
|  | 1. ঠান্ডা লাগা feels cold | | | | | | | D |  |
|  | 1. খেতে না পারা বা বুকের দুধ টানতে না পারা Inability to feeding or stopped feeding | | | | | | | E |  |
|  | 1. চামড়া হলুদ হয়ে যাওয়া Yellow skin | | | | | | | F |  |
|  | 1. অন্যান্য (নির্দিষ্ট করুন) Other (specify)_____________________ | | | | | | | Y |  |
| E9 | প্রসবের পরে স্বাস্থ্যকর্মী কি আপনার বাচ্চাকে পরীক্ষা করেছেন? Did a health worker examine your baby after delivery | হ্যাঁ Yes | | | | | | 1 |  |
|  |  | না No | | | | | | 2 |  |
|  |  | মৃত জন্ম/ প্রযোজ্য নয় | | | | | | 3 | 🡪 E11 |
|  |  | জানা নেই Don’t know | | | | | | 8 |  |
| E10 | প্রসবের পর আপনার বাচ্চাকে যে পরিমাণ যত্ন এবং মনোযোগ দেওয়া হয়েছে তা নিয়ে কি আপনি সন্তুষ্ট? Are you satisfied with the level of attention and care given to your newborn baby after delivery? | | | | 1 | | | 2 |  |
| E11 | প্রসবের পর কোন স্বাস্থ্যসেবাদানকারী কি আপনাকে পরীক্ষা করেছে? Did health workers examine you at any time after delivery | | | | 1 | | | 2 |  |
| E12 | বাচ্চাকে স্বাস্থ্যকেন্দ্র থেকে ছাড়ার পূর্বে তাকে কি মাথা থেকে পা পর্যন্ত পরীক্ষা করা হয়েছিল? Was the baby examined from head to toe before discharge? | হ্যাঁ Yes | | | | | | | 1 |
|  |  | না No | | | | | | | 2 |
|  |  | মৃত জন্ম/ প্রযোজ্য নয় | | | | | | | 3 |
| E13 | আপনি কি স্বাস্থ্যসেবাদানকারীর ব্যবহার নিয়ে সন্তুষ্ট? Are you satisfied about the attitude of the health worker(s)? | | | | 1 | | | 2 |  |
| E14 | আপনাকে কি আপনার কোন সমস্যা নিয়ে প্রশ্ন করার সুযোগ দেওয়া হয়েছিল? Were you given the opportunity to ask questions or express your concerns? | | | | 1 | | | 2 |  |
| E15 | আপনার কি মনে হয় স্বাস্থ্যকর্মী আপনি এখানে থাকাকালীন সময়ে সেবা দেওয়ার ক্ষেত্রে আপনার সুবিধা অসুবিধা ভেবেছেন? Do you think health workers took your concerns into consideration in providing care during your stay there? | | | | 1 | | | 2 |  |

1. **Hygiene and Hand washing :**

| **No** | **Points & questions** | **Options** | **Code** | **skip** |
| --- | --- | --- | --- | --- |
| F1 | লেবার ওয়ার্ডে কি বাথরুমের ব্যবস্থা ছিল? Is there Toilet in the labour ward? | হ্যাঁ Yes | 1 |  |
|  |  | না No | 2 | **🡪** F3 |
|  |  | জানা নেই Don’t know | 8 | **🡪** F3 |
| F2 | আপনি কি তা ব্যবহার করেছিলেন? Did you use it | হ্যাঁ Yes | 1 |  |
|  |  | না No | 2 |  |
| F3 | মা ও শিশু ওয়ার্ডে কি বাথরুম আছে? Is there a toilet for patients in the maternity or children’s ward? | হ্যাঁ Yes | 1 |  |
|  |  | না No | 2 |  |
|  |  | জানা নেই Don’t know | 8 |  |
| F4 | বাথরুমের পরিবেশ সম্পর্কে আপনার মতামত কি? What do you think of the toilet? | অনেক পরিষ্কার very clean | 1 |  |
|  |  | মোটামুটি পরিষ্কার/ চলে just okay | 2 |  |
|  |  | অপরিষ্কার unclean | 3 |  |
|  |  | অনেক অপরিষ্কার very unclean | 4 |  |
| F5 | আপনি যতক্ষণ লেবার ওয়ার্ডে ছিলেন ততক্ষন কি আপনার পানি ব্যবহার করা এবং সাবান দিয়ে হাত ধোয়ার সুযোগ ছিল? During your stay in ward did you have access to water & soap to wash your hand | হ্যাঁ Yes | 1 |  |
|  |  | না No | 2 |  |
| F6 | স্বাস্থ্যকেন্দ্রে থাকাকালীন সময়ে আপনি কি একবারো হাত ধুয়েছিলেন? Did you wash your hand while you stayed at facility**?** | হ্যাঁ Yes | 1 |  |
|  |  | না No | 2 | **🡪** F8 |
| F7 | হাত ধোয়ার পর তা শুকিয়েছেন কিভাবে? How did you dry your hands after washing? | বাতাসে শুকিয়েছি air dry | 1 |  |
|  |  | পরিষ্কার কাপড়/ তোয়ালে দিয়ে with clean towels | 2 |  |
|  |  | অপরিষ্কার কাপড়/ তোয়ালে দিয়ে with unclean towel | 3 |  |
| F8 | সব মিলিয়ে স্বাস্থ্যকেন্দ্রের পরিষ্কার পরিচ্ছন্নতা নিয়ে আপনার মত কি? Overall, how satisfied were you with the hygiene standards at the health facility? | অনেক পরিষ্কার very clean | 1 |  |
|  |  | মোটামুটি পরিষ্কার/ চলে just okay | 2 |  |
|  |  | অপরিষ্কার unclean | 3 |  |
|  |  | অনেক অপরিষ্কার very unclean | 4 |  |

1. **Payment for services:**

| **No** | **Points & questions** | **Options** | | **Coding** | **skip** |
| --- | --- | --- | --- | --- | --- |
| G1 | লেবার বা প্রসব সংক্রান্ত কারণে আপনা কি কোন খরচ করতে হয়েছে? (টাকা বা অন্যকিছু) Did you have to pay anything (cash or kind) for the service provided during labour or delivery | হ্যাঁ Yes | | 1 |  |
|  |  | না No | | 2 |  |
| G2 | আপনাকে কি আপনার নিজের অথবা বাচ্চার জন্য কোন কিছু কিনতে বলা হয়েছিলো? Were you asked to purchase some items for yourself or for baby? | হ্যাঁ Yes | | 1 |  |
|  |  | না No | | 2 |  |
| G3 | নিম্নোক্ত বিষয়গুলোর জন্যে আপনাকে কত টাকা খরচ করতে হয়েছে? (কোনো খরচ না হলে “০০০০০” লিখুন) How much did you pay in total for the following? *(write “00000” if no payment needed)* | | | |  |
|  | 1. স্বাস্থ্যকেন্দ্রে থাকতে *Sleeping in facility* | | টাকা taka | |  |
|  | 1. সরঞ্জাম কিনতে *Supplies* | | টাকা taka | |  |
|  | 1. ওষুধ কিনতে *Drugs* | | টাকা taka | |  |
|  | 1. আল্ট্রাসাউন্ড করতে *Ultrasound scan* | | টাকা taka | |  |
|  | 1. ডায়াগনস্টিক পরীক্ষা করতে *Laboratory investigation* | | টাকা taka | |  |
|  | 1. স্বাস্থ্যকেন্দ্রের খাবার খেতে *Food provided by facility* | | টাকা taka | |  |
|  | 1. প্রসবের জন্যে *Delivery care* | | টাকা taka | |  |
|  | 1. অন্যান্য (নির্দিষ্ট করুন) *Other (specify)*   *_____________________________________* | | টাকা taka | |  |

1. **Overall impressions about the care in the facility**

| **No** | **Points & questions** | **Options** | **Coding** | **skip** |
| --- | --- | --- | --- | --- |
| H1 | ভবিষ্যতে কোনদিন যদি আপনি আবারো গর্ভবতী হন, তবে কি এখানে ডেলিভারী করাতে আসবেন? If you have to deliver again will you come back to this health facility? | অবশ্যই definitely | 1 |  |
|  |  | মনে হয় maybe | 2 |  |
|  |  | যদি খুব প্রয়োজন না হয় if I can't help it | 3 |  |
|  |  | না, কখনোই না No, never | 4 |  |
| H2 | আপনি কি আপনার কোন আত্মীয় বা বন্ধু কে এখানে ডেলিভারী করাতে বলবেন? Would you recommend this health facility to a relative or friend for them to deliver? | হ্যাঁ Yes | 1 |  |
|  |  | না No | 2 |  |
| H3 | এই স্বাস্থ্যকেন্দ্রে ডেলিভারী করাতে এসে সেবা পেয়ে আপনি সামগ্রিক ভাবে কেমন সন্তুষ্ট? In general, how satisfied are you with the care you received during your delivery at this health facility?  *(read out the options)* | বেশ সন্তুষ্ট very satisfied | 1 |  |
|  |  | সন্তুষ্ট satisfied | 2 |  |
|  |  | মোটামুটি neutral | 3 |  |
|  |  | অসন্তুষ্ট unsatisfied | 4 |  |
|  |  | বেশ অসন্তুষ্ট very unsatisfied | 5 |  |
| H4 | সামগ্রিক ভাবে কোন জিনিষগুলো আপনার ভালো লেগেছে? In general what did you like | **----------------------------------------** | |  |
| H5 | সামগ্রিক ভাবে কোন জিনিষগুলো আপনার ভালো লাগেনি? What did you not like | **----------------------------------------** | |  |
| H6 | আপনাকে এখানকার কোন কিছু যদি পরিবর্তন বা উন্নতি করতে বলা হয় আপনি কি কি করবেন? If you could change anything what would you recommend? | **----------------------------------------** | |  |

1. **Questions for Family Member**

| I1 | আপনার বন্ধু/ আত্মীয়/ স্ত্রী এখানে যে লেবারের ও সন্তান প্রসবের জন্যে আসলেন এবং সেবা পেলেন তা আপনার কাছে কেমন মনে হয়েছে? How would you describe the care your relative/friend/wife received during labour and delivery at this facility?  ___________________________________________________________________________ |
| --- | --- |
| I2 | এখানে থাকা অবস্থায় আপনি কি কোন ধরণের অনৈতিক আচরনের সম্মুখীন হয়েছেন? যদি হয়ে থাকেন, তবে বলুন কি হয়েছিল? Did you face any kind of abuse during your stay at this facility while your relative/friend/wife was in labour? If Yes explain the kind of abuse _____________________________________  ___________________________________________________________________________  ___________________________________________________________________________ |
| I3 | এখানে থাকা অবস্থায় আপনার বন্ধু/ আত্মীয়/ স্ত্রী যিনি এখানে লেবারের ও সন্তান প্রসবের জন্যে আসলেন তিনি কি কোন ধরণের অনৈতিক আচরনের সম্মুখীন হয়েছেন? যদি হয়ে থাকেন, তবে বলুন কি হয়েছিল? In your stay to this facility, did you observe your relative/friend/wife being abused in any way during her labour and delivery? If Yes, explain what was done to her ___________________________________________________________________________  ___________________________________________________________________________ |
| I4 | এই স্বাস্থ্যকেন্দ্রে আপনার বন্ধু/ আত্মীয়/ স্ত্রী ডেলিভারী করাতে য়াসলেন তা নিয়ে আপনি সামগ্রিক ভাবে কেমন সন্তুষ্ট? In general, how satisfied are you with the care provided to your relative/ friend/wife during her labour and delivery at this health facility?  (read out the options): বেশ সন্তুষ্ট very satisfied; সন্তুষ্ট satisfied ; মোটামুটি neutral; অসন্তুষ্ট unsatisfied; বেশ অসন্তুষ্ট very unsatisfied  ___________________________________________________________________________ |
| I5 | সামগ্রিক ভাবে কোন জিনিশ গুলো আপনার ভালো লেগেছে? What did you like most in this facility and why?  ___________________________________________________________________________  ___________________________________________________________________________ |
| I6 | সামগ্রিক ভাবে কোন জিনিশ গুলো আপনার ভালো লাগেনি? What did you not like  ___________________________________________________________________________  ___________________________________________________________________________ |
| I7 | আপনাকে এখানকার কোন কিছু যদি পরিবর্তন করতে বলা হয় আপনি কি কি করবেন? If you could change anything what would you recommend?  ___________________________________________________________________________  ___________________________________________________________________________ |

N.B: Thank you for giving us your valuable time. ধন্যবাদ আমাদের আপনার মূল্যবান সময় দেওয়ার জন্যে।

**Time of ending of interview:** : **(24 hour)**
